# Capturing a methanogenic carbon monoxide dehydrogenase/acetyl-CoA synthase complex via cryogenic electron microscopy

**DOI:** 10.1073/pnas.2410995121

**Published:** 2024-10-03

**Authors:** Alison Biester, David A. Grahame, Catherine L. Drennan

**Affiliations:** ^a^Department of Chemistry, Massachusetts Institute of Technology, Cambridge, MA 02139; ^b^Department of Biochemistry and Molecular Biology, Uniformed Services University of the Health Sciences, Bethesda, MD 20814; ^c^Department of Biology, Massachusetts Institute of Technology, Cambridge, MA 02139; ^d^HHMI, Massachusetts Institute of Technology, Cambridge, MA 02139

**Keywords:** greenhouse gases, methanogenesis, gas channels, cryogenic electron microscopy

## Abstract

Approximately two-thirds of the estimated one-billion metric tons of methane produced annually by methanogens is derived from the cleavage of acetate. Acetate is broken down by a Ni-Fe-S-containing A-cluster within the enzyme acetyl-CoA synthase (ACS) to carbon monoxide (CO) and a methyl group (CH_3_^+^). The methyl group ultimately forms the greenhouse gas methane, whereas CO is converted to the greenhouse gas carbon dioxide (CO_2_) by a Ni-Fe-S-containing C-cluster within the enzyme carbon monoxide dehydrogenase (CODH). Although structures have been solved of CODH/ACS from acetogens, which use these enzymes to make acetate from CO_2_, no structure of a CODH/ACS from a methanogen has been reported. In this work, we use cryo-electron microscopy to reveal the structure of a methanogenic CODH and CODH/ACS from *Methanosarcina thermophila* (*Met*CODH/ACS). We find that the N-terminal domain of acetogenic ACS, which is missing in all methanogens, is replaced by a domain of CODH. This CODH domain provides a channel for CO to travel between the two catalytic Ni-Fe-S clusters. It generates the binding surface for ACS and creates a remarkably similar CO alcove above the A-cluster using residues from CODH rather than ACS. Comparison of our *Met*CODH/ACS structure with our *Met*CODH structure reveals a molecular mechanism to restrict gas flow from the CO channel when ACS departs, preventing CO escape into the cell. Overall, these long-awaited structures of a methanogenic CODH/ACS reveal striking functional similarities to their acetogenic counterparts despite a substantial difference in domain organization.

## Significance

Carbon monoxide dehydrogenase (CODH) and acetyl-CoA synthase (ACS) play major roles in the global carbon cycle through the consumption of CO and CO_2_ gases, production of acetate, and breakdown of acetate for methane generation. These enzymes are proposed to have been present in the last universal common ancestor based on sequence conservation of CODH and ACS between bacterial and archaeal domains of life. To complement published structural studies of bacterial CODH/ACS complexes, here we present the structure of an archaeal CODH/ACS complex and discuss intriguing similarities between bacterial and archaeal CODH/ACS enzymes. This work provides insights into the common features of CODH/ACS that were likely present in the enzymes utilized by Earth’s earliest life forms.

Acetogens and methanogens are theorized to be the first life forms because they are capable of autotrophic growth on CO_2_ and H_2,_ gases thought to be abundant in the early atmosphere (1, 2). These microbes can synthesize acetate, a basic building block of life, from two molecules of CO_2_ with reducing equivalents derived from H_2_. A key enzyme involved is a 310 kDa bifunctional carbon monoxide dehydrogenase (CODH)/acetyl-CoA synthase (ACS), which utilizes multimetal ion cofactors composed of nickel, iron, and sulfur (3). Conservation in both bacterial and archaeal domains of life suggests that CODH and ACS are among the oldest enzymes and were present in the last universal common ancestor (LUCA) (1, 4). In the modern global carbon cycle, acetogens and methanogens contribute approximately 10^9^ tons of acetate and 10^9^ tons of methane, respectively, each year (5, 6).

**Scheme 1.**
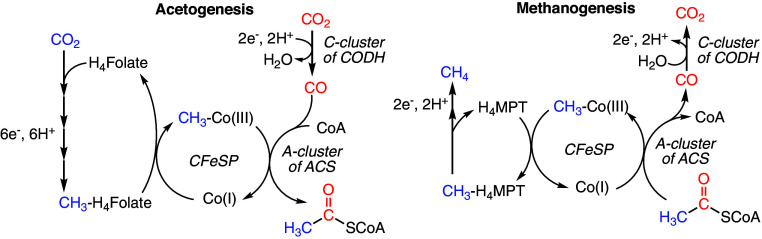
Pathways of acetogenesis and methanogenesis.

CODH of acetogenic CODH/ACS uses a Ni–Fe–S-containing C-cluster to reversibly reduce CO_2_ to CO, the biological equivalent of the water-gas shift reaction (Scheme 1 and *SI Appendix*, Fig. S1*A*). The generated CO travels through a gas channel (7, 8) within CODH/ACS to the Ni–Fe–S-containing A-cluster of ACS where it is combined with a methyl moiety and coenzyme A to make acetyl-CoA, in the biological equivalent of the Monsanto process (Scheme 1 and *SI Appendix*, Fig. S1*A*). In acetogens, the methyl group is delivered to the A-cluster by an 83 kDa corrinoid iron-sulfur protein (CFeSP), using its Co-containing corrinoid cofactor (Scheme 1 and *SI Appendix*, Fig. S1 *A* and *D*). Thus, acetyl-CoA production requires both a bifunctional CODH/ACS and a standalone CFeSP (9, 10). By contrast, methanogenic archaea employ four copies of CODH (α_2_ε_2_ heterotetramers) and eight copies of ACS (β monomers) that interact stably with eight copies of CFeSP (γδ heterodimers) to form a massive 2.2 MDa complex called the acetyl-CoA decarbonylase/synthase (ACDS) complex (*SI Appendix*, Fig. S1*B*) (11–13). Approximately two-thirds of the estimated one-billion metric tons of methane produced annually by methanogens on Earth is derived from the cleavage of acetate (5, 14, 15). ACDS plays a central role in that process, wherein the A-cluster of ACS cleaves acetyl-CoA into CO and a methyl moiety, the C-cluster of CODH oxidizes CO to CO_2_, and the corrinoid of CFeSP receives the methyl moiety, which is reduced to methane in downstream methanogenic metabolism (Scheme 1 and *SI Appendix*, Fig. S1*B*). However, archaea can also use ACDS for acetyl-CoA synthesis, which was likely the way ACDS was used in early archaeal metabolism (16, 17). Finally, in the absence of ACS, CODH can couple CO oxidation to hydrogen production (*SI Appendix*, Fig. S1*C*) (18). Such monofunctional CODHs are believed to have originated early in bacterial evolution by gene duplication from the CODH of a bifunctional CODH/ACS complex (4). These enzymes are estimated to eliminate approximately 10^8^ tons of CO from the atmosphere annually (19).

Monofunctional CODHs were the first to be structurally characterized (20, 21). They were found to be homodimeric, where each monomer consists of an N-terminal helical domain and two Rossmann folds (*SI Appendix*, Fig. S2*A*). Each homodimer contains five metalloclusters (*SI Appendix*, Fig. S1*D*), including one 4Fe–4S B-cluster per monomer and one 2Fe–2S or 4Fe–4S D-cluster bridging the monomers, all of which are bound by the N-terminal helical domain (20–24). The D- and B-clusters are responsible for transferring electrons to and from the catalytic Ni–Fe–S C-cluster, which is sandwiched between the two Rossmann folds in each monomer.

The structure of a bifunctional CODH/ACS heterotetramer from the acetogen *Moorella thermoacetica* (*Moor*) (25) revealed the use of N- and C-terminal extensions from a classic CODH core fold to bind ACS, which has a three-domain structure (*SI Appendix*, Fig. S2 *B* and *C*). The N-terminal domain of ACS, termed A1, is composed of a helical bundle preceding a Rossmann fold. The central domain and C-terminal domains, termed A2 and A3, respectively, are both α/β domains. The A3 domain binds the catalytic Ni–Fe–S A-cluster. More recently, another acetogenic CODH/ACS from *Clostridium autoethanogenum* (*Ca*) was structurally characterized, revealing an enzyme with the same overall fold, but a dramatically different interface between CODH and ACS (*SI Appendix*, Fig. S2*C*) (26). The *Ca*CODH does not contain the N- and C-terminal extensions found in *Moor*CODH, and instead binds ACS through a loop and helix at the N-terminal end of Rossmann fold 2 (helix αA and preceding loop, *SI Appendix*, Fig. S2 *A* and *C*). In both *Ca* and *Moor*CODH/ACS, it is the A1 domain of ACS used to bind CODH (*SI Appendix*, Fig. S2 *B* and *C*).

Much less is known about the structures of the methanogenic ACDS proteins, the focus of this study. Only one component, the methanogenic CODH heterotetramer as isolated from an ACDS complex, has been structurally characterized (*SI Appendix*, Fig. S3*A*) (27, 28). The N-terminal helical domain, Rossmann folds 1 and 2, and the B-, C-, and D-clusters of the monofunctional and acetogenic CODH are also present in the methanogenic CODH from *Methanosarcina barkeri* (*Meb*); however, the methanogenic CODH has several key differences in its fold and has additional chains, domains, and cofactors. The additional chain in methanogenic CODH is called the ε subunit, in which a single Rossmann fold is the major structural element; this chain binds the α subunit and buries the D-cluster. Between the two Rossmann folds of the α subunit, there is an iron-sulfur cluster domain insert harboring two additional 4Fe–4S clusters, the E- and F-clusters; the F-cluster is surface exposed and acts as the entry point for transferring electrons to and from the C-cluster active site. Finally, the α subunit of the methanogenic CODH contains a C-terminal extension. From sequence information we know that this ACS lacks the A1 domain but has A2 and A3 domains conserved with its monofunctional and acetogenic ACS counterparts, along with a unique C-terminal extension (*SI Appendix*, Fig. S3*B*).

The absence of the A1 domain in methanogenic ACS raises several questions. First, in acetogens, A1 serves as the binding domain for CODH, so it is unclear how ACS binds to CODH in methanogens. Second, the gas channel that carries CO from the C-cluster in CODH to the A-cluster in acetogenic CODH/ACSs (*SI Appendix*, Fig. S4*A*) is a major component contained in the A1 domain, which raises the question of how CO transits between the clusters in methanogens (28). Third, A1 of *Moor*CODH/ACS contributes residues to the upper face of the A-cluster, forming an alcove for a cluster-bound CO molecule when the gas channel is open (*SI Appendix*, Fig. S4*A*, inset) (29, 30). Whether or not a similar CO alcove exists in methanogen ACS, and how similar or different it might be, has been a subject of speculation and motivation for structural studies.

Important protein conformational changes are implicated in the CODH/ACS mechanism. *Moor*ACS must open up to expose the otherwise buried A-cluster to allow for methylation by CFeSP. Evidence for this movement has been obtained from both crystallography (29, 31) and by negative stain EM (32), which involves swinging of A2 and/or A3 away from A1 (*SI Appendix*, Fig. S4*B*), and also, the closing of the gas channel by movement of helix αA of A1 (*SI Appendix*, Fig. S4*B*, inset) (31). Thus, at least two different major conformations exist for *Moor*CODH/ACS, an open-channel/closed ACS for A-cluster carbonylation and a closed-channel/open ACS for A-cluster methylation. For both states, the A1 domain of ACS plays a key role, but how these dynamic changes are accomplished in methanogens in the absence of A1 is entirely unknown.

In this study, we investigate the structure of methanogenic CODH and CODH/ACS complexes from *Methanosarcina thermophila* (*Met*). Through plunge freezing of the ACDS complex, we captured multiple subcomplexes including a CODH tetramer, a CODH/ACS pentamer, and a CODH/ACS hexamer and characterized these subcomplexes via cryogenic electron microscopy (cryo-EM). These structures reveal a CODH/ACS interface that differs from the interface observed in acetogens but displays a remarkably similar CO-binding alcove. We observe a hydrophobic cavity between the C- and A-clusters in the CODH/ACS complex that is likely used for gas transport, and notably is much shorter than the channel in *Moor*CODH/ACS. In comparing the CODH alone with the CODH/ACS complex, we have identified a molecular mechanism to close the gas channel when ACS is absent. This molecular mechanism is like that of *Moor*CODH/ACS. The results contribute a substantial advance to the understanding of key structural and functional similarities and differences between the acetogenic and methanogenic CODH/ACS interactions.

## Results

### Sample Preparation on Next-generation Automated Chameleon Plunging Instrument Yields CODH/ACS Subcomplexes.

**Fig. 1.**
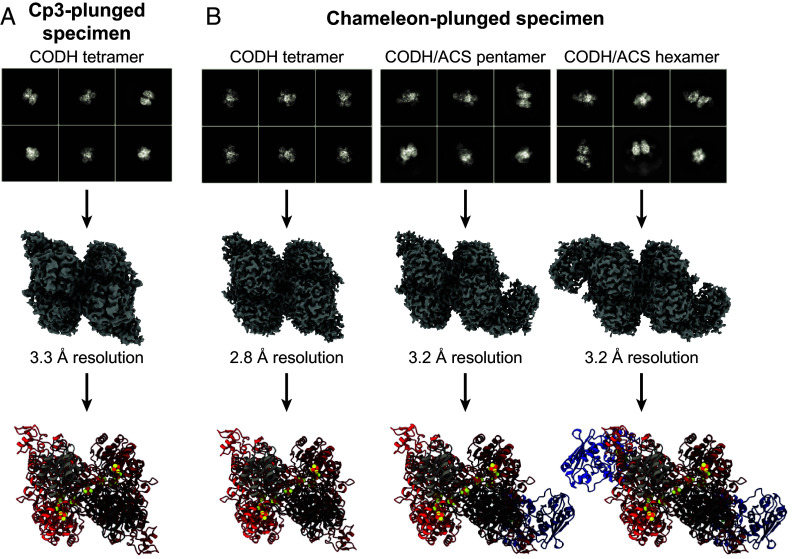
Traditional EM grid blot-plunging yields CODH tetramer particles, whereas chameleon-plunging yields CODH tetramer, CODH/ACS pentamer, and CODH/ACS hexamer subcomplexes. (*A*) 2D classes, final EM map, and model of CODH tetramer particles from Cp3-plunged specimen. (*B*) 2D classes, final EM maps, and models of CODH tetramer, CODH/ACS pentamer, and CODH/ACS hexamer from chameleon-plunged specimen. CODH α subunits are colored in red, CODH ε subunits are colored in gray, and ACS β subunits are colored in blue.

Previous work on the methanogenic ACDS complex has shown that subcomponents can be isolated using treatments with detergent or limited proteolysis, along with ion exchange chromatography (33–35). Through these methods, the α_2_ε_2_ CODH subunit (33–35), a truncated form of the β ACS subunit (33, 34), and the γδ CFeSP subunit (30) have each been isolated, as well as a β(γδ) ACS/CFeSP subcomplex (34). However, isolation and characterization of an (α_2_ε_2_)β CODH/ACS subcomplex has not been reported. In this work, all experiments were performed using intact *Met*ACDS complex, and on-grid dissociation allowed for visualization of subcomponents through cryo-EM. Using a traditional blot-plunging method, α_2_ε_2_ CODH subcomplexes were observed (Fig. 1*A* and *SI Appendix*, Table S1). Sufficient CODH subcomplex was present on the grid to generate a high-resolution reconstruction, however, in 2D and 3D classifications of CODH particles, there were no classes containing additional density consistent with ACS (*SI Appendix*, Figs. S5 and S6). With the next-generation chameleon instrument (36, 37), different dissociation behavior led to formation of (α_2_ε_2_)β and (α_2_ε_2_)β_2_ CODH/ACS subcomplexes in addition to the α_2_ε_2_ CODH subcomplexes, allowing us to capture methanogenic CODH/ACS subcomplexes (Fig. 1*B* and *SI Appendix*, Figs. S7–S9 and Table S1). The chameleon technology, based on Spotiton (38, 39), is intended to ameliorate issues of particle denaturation, and it is therefore unsurprising that we observe differences in dissociation behavior relative to the blot-plunge method. Findings using the chameleon instrument have demonstrated that short dispense-to-plunge times (<300 ms) can help mitigate particle denaturation (40, 41), and another study showed that the chameleon can yield transient protein oligomeric states in sufficient abundance for structure determination, which was not true when using blot-plunging (42). Here, we observe a similar phenomenon, wherein the full 2.2 MDa ACDS complex is dissociated to a lesser extent on the chameleon than on a blot-plunger; in both the blot-plunged specimen and the chameleon-plunged specimen, CODH particles were observed, but CODH/ACS subcomplexes were only observed in the chameleon-plunged specimen, suggesting that the ACDS complex underwent more dissociation under blot-plunging conditions. The use of two plunging methods allowed us to capture two snapshots of *Met*CODH, with global resolutions of 2.8 and 3.3 Å for the maps from chameleon and Cp3-plunged samples, respectively (Fig. 1 *A* and *B* and *SI Appendix*, Figs. S6 and S10). Additionally, from the chameleon-plunged specimen, structures of the *Met*CODH/ACS pentamer and hexamer were determined, each at a global resolution of 3.2 Å (Fig. 1*B* and *SI Appendix*, Figs. S11 and S12).

### Unique CODH/ACS Interface Is Observed in Methanogens.

**Fig. 2.**
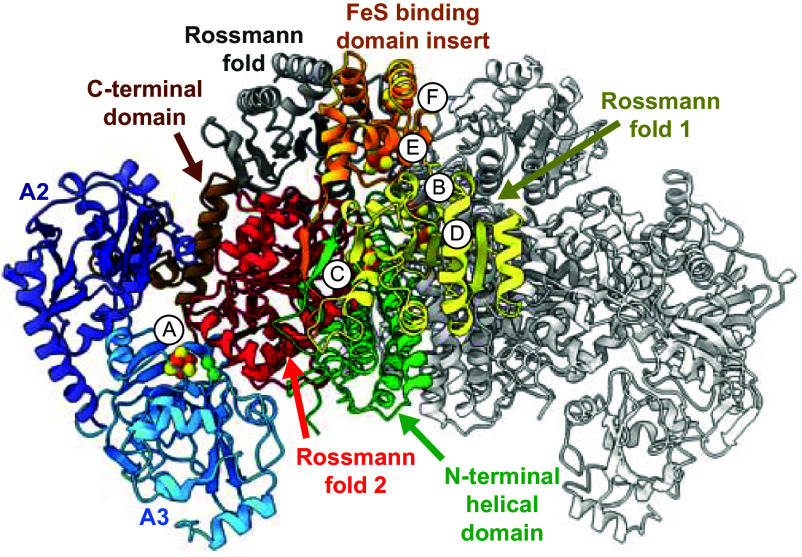
CODH/ACS hexameric complex from *M. thermophila*. The locations of domains and cofactors are indicated.

The *Met*CODH α_2_ε_2_ heterotetramer shares the same topology as *Meb*CODH (Fig. 2 and *SI Appendix*, Figs. S3*A* and S13*A*) and harbors the same set of metallocofactors, including E- and F-clusters, which are not present in monofunctional and acetogenic CODHs (*SI Appendix*, Figs. S13 *B* and *C* and S14 *B*–*F*) (28). As expected based on the amino acid sequence (43, 44), *Met*ACS shares the topology as the A2 and A3 domains of monofunctional and acetogenic ACSs, but lacks the N-terminal A1 domain (Fig. 2 and *SI Appendix*, Figs. S2*B*, S3*B*, and S13*A*). Although *Met*ACS contains an ~75 amino acid C-terminal extension of unknown function, that extension could not be resolved in our EM data and is likely highly flexible. To facilitate comparisons with its acetogenic counterparts, the two ACS domains of *M. thermophila* are referred to here as the A2 (central domain, residues 4 to 151) and A3 (C-terminal domain, residues 186 to 397).

The structures reported here reveal that both A2 and A3 of ACS contact CODH (Fig. 2). This interface does not bear resemblance to any previously observed CODH/ACS interface; all reported CODH/ACS structures to date are from acetogenic bacteria, wherein it is solely A1 of ACS that interacts with CODH (*SI Appendix*, Fig. S13*A*). On CODH, the interaction surface is formed largely by Rossmann fold 2 (Fig. 2). Interestingly, the C-terminal extension of *Met*CODH, which differs from the C terminus of non-methanogenic CODHs, interacts with ACS. The *Met*CODH/ACS interface is also distinct because this interaction includes direct packing of the A-cluster against CODH.

### CO-Binding Alcove in Acetogens Is Conserved in Methanogens.

**Fig. 3.**
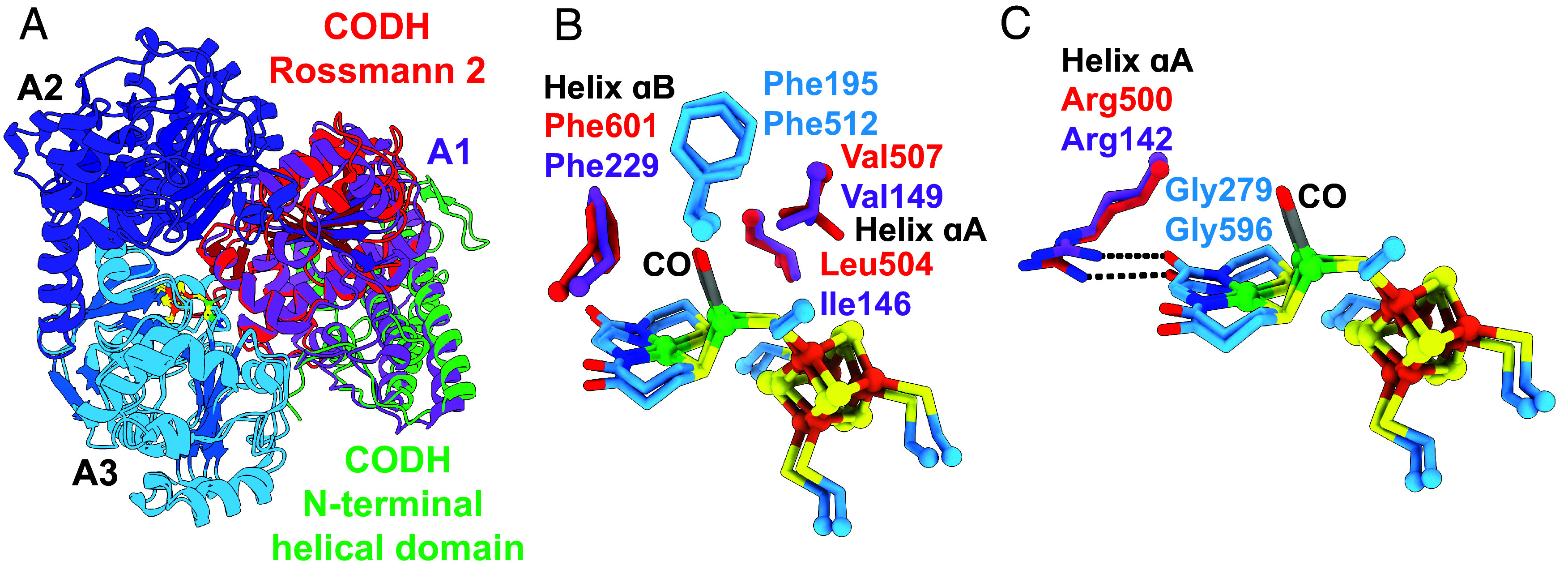
*Met*CODH Rossmann fold 2 takes the place of *Moor*ACS domain 1 and creates a CO alcove above Ni_p_ in the A-cluster. (*A*) Overlay of *Met*CODH (red and green)/ACS (cyan and blue) with the closed conformation of *Moor*ACS (PDBID: 6X5K) (A1 in purple). For clarity, only Rossmann fold 2 (red) and the N-terminal helical domain (green) of *Met*CODH are shown. (*B*) Conservation of residues in the CO alcove of the A-cluster between *Met*CODH/ACS and *Moor*ACS. (*C*) Conservation of arginine: A-cluster interactions. The A-cluster is colored: Ni green; Fe orange; S yellow; CO gray/red. Carbons are colored by domains as in Fig. 2.

In the structure of *Met*CODH/ACS, we observe density consistent with CO binding to the proximal nickel of the A-cluster (*SI Appendix*, Fig. S14*A*). The geometry of Ni_p_ is tetrahedral as observed previously for a CO-bound A-cluster (29). Superimposition with the CO-bound *Moor*CODH/ACS structure (aligned with respect to A3 of ACS) reveals several similarities despite the interface differences. Where in *Moor*ACS A1 packs against A3, in *Met*CODH/ACS we find Rossmann fold 2 of CODH packed against A3 of ACS in an analogous fashion, and the helices preceding the Rossmann fold of *Moor*ACS align with the N-terminal helical domain of *Met*CODH (Fig. 3*A* and *SI Appendix*, Fig. S13 *D* and *E*). The environment surrounding the CO-bound A-cluster of *Met*CODH/ACS is remarkably similar to that of *Moor*CODH/ACS. In *Moor*CODH/ACS, four second-sphere residues have been noted as important for forming the CO-binding alcove: Ile146 and Val149 on helix αA and Phe229 on helix αB from A1, and Phe512 from A3 (29, 30). Phe512 of *Moor*ACS is conserved in the A3 domain, corresponding to Phe195 in *Met*ACS (Fig. 3*B* and *SI Appendix*, Figs. S15 and S16*A*). Substituting for Ile146, Val149, and Phe229 of the A1 domain of *Moor*ACS are Leu504, Val507, and Phe601 of Rossmann fold 2 in *Met*CODH, respectively (Fig. 3*B* and *SI Appendix*, Figs. S15 and S16*B*). An interaction made to an A-cluster coordinating residue is also conserved. Arg142 of the A1 domain of *Moor*ACS corresponds to Arg500 of Rossmann fold 2 in *Met*CODH. Both arginine residues hydrogen bond with the carbonyl of a glycine whose backbone nitrogen coordinates Ni_d_ (Fig. 3*C*).

### The CO Channel for Methanogenic CODH/ACS Is Also Observed in Monofunctional CODH but Differs From Paths in Acetogenic CODH/ACSs.

**Fig. 4.**
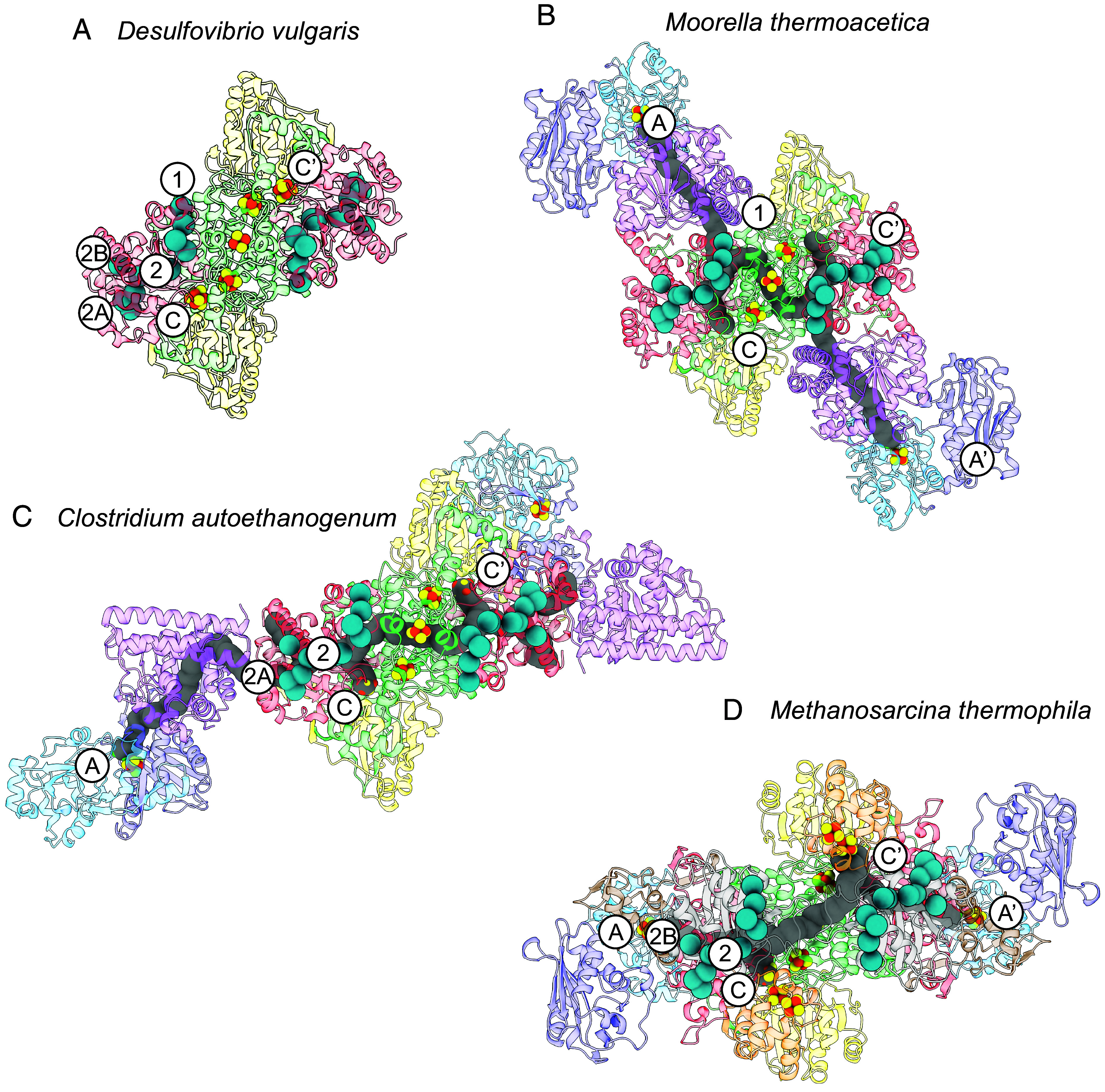
Gas channels from monofunctional CODH compared with acetogenic and methanogenic CODH channels. (*A*) The monofunctional CODH from *D. vulgaris* has three gas channels (1, 2A, and 2B) elucidated by xenon studies (PDBID: 7TSJ). (*B*) Structure of *M. thermoacetica* CODH/ACS (PDBID: 6X5K) with calculated cavities. Xe sites from *D. vulgaris* CODH are overlaid. Channel 1, which is extended to form the CO gas channel spanning from the C- to A-cluster for the acetogenic CODH/ACS from *M. thermoacetica*. The C- and A-clusters, which are ~70 Å apart in *M. thermoacetica*, are connected by a channel that is ~110 Å long. (*C*) Structure of *C. autoethanogenum* CODH/ACS (PDBID: 6YTT) with calculated cavities. Xe sites from *D. vulgaris* CODH are superimposed. Channels 2 and 2A align with the CO gas channel leading from the C-cluster to ACS in the acetogenic CODH/ACS from *C. autoethanogenum*. (*D*) Structure of *M. thermophila* CODH/ACS (this work) with calculated cavities. Xe sites from *D. vulgaris* CODH are overlaid. Channels 2 and 2B align with the CO gas channel leading from the C- to A-cluster for the methanogenic CODH/ACS from *M. thermophila*. The C- and A-clusters in *M. thermophila*, which are ~35 Å apart, are connected by a channel that is ~50 Å long. Xe sites are shown as cyan spheres. Cavities were calculated using MOLEonline, and calculated cavities are shown in gray. Proteins are colored as in Fig. 2. See *SI Appendix*, Fig. S17 for calculated cavities without Xe sites superimposed.

Xenon gas has been used to map gas channels in the monofunctional CODH from *Desulfovibrio vulgaris* (45). Two channels were identified, with channel 1 traversing from near the C-cluster to the enzyme surface, whereas channel 2 branches off into channels 2A and 2B, with each branch leading to the enzyme surface (Fig. 4*A* and *SI Appendix*, Fig. S17*A*). Channel 1 from the monofunctional CODH aligns with the C- to A-cluster gas channel in *Moor*CODH/ACS (Fig. 4*B* and *SI Appendix*, Fig. S17*B*) as elucidated via xenon gassing studies (46) and observed in cavity calculations. Structural analysis shows that the distance between the C- and A-cluster in *Moor*CODH/ACS is ~70 Å, with the cavity between them weaving through the protein along a path that totals ~110 Å in length, based on cavity calculations. For *Ca*CODH/ACS, which displays a different ACS:CODH interface (26), the gas channel is rerouted relative to the *Moor* enzyme (based on cavity calculations). The channel in *Ca*CODH is shared with channels 2 and 2A in the monofunctional CODH (Fig. 4*C* and *SI Appendix*, Fig. S17*C*). Upon performing cavity calculations on the *Met*CODH/ACS complex, we see that this enzyme uses channel 2, like *Ca*CODH/ACS, but *Met*CODH/ACS uses the channel 2B branch (Fig. 4*D* and *SI Appendix*, Fig. S17*D*). Given that *Met*ACS lacks A1, the ACS packs in more tightly to the CODH compared to the acetogenic CODH/ACS complexes, giving rise to a much shorter distance between the C- and A-clusters, with a measured distance of ~35 Å, and the internal cavity totaling ~50 Å in length. It is intriguing that all three channels observed in the monofunctional CODH are present in the distinct CODH/ACSs whose structures have been determined to date.

### Conformational Changes in CODH Facilitate Opening and Closing of the CO Channel.

**Fig. 5.**
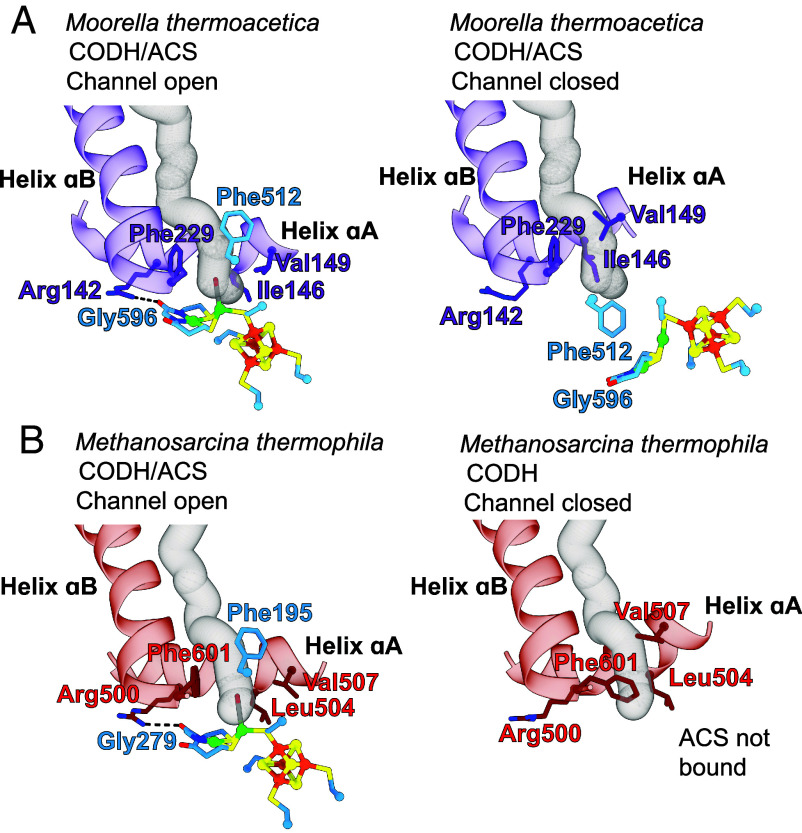
Movement of conserved Rossmann helices caps the gas channel in acetogens and methanogens. (*A*) In the open-channel/closed-ACS conformation of *Moor*ACS (PDBID: 6X5K), the gas channel passes between helices αA and αB, with alcove residues surrounding the CO site and Arg142 hydrogen bonding to an A-cluster coordinating residue (Gly596). In the closed-channel/open-ACS conformation of *Moor*ACS (PDBID: 1OAO), helix αA blocks the gas channel, Phe512 blocks the CO-binding site at the A-cluster, and Arg142 has swung away from the A-cluster breaking its hydrogen bond. (*B*) In *Met*CODH/ACS, the gas channel also passes between helices αA and αB, with alcove residues surrounding the CO site and Arg500 hydrogen bonding to an A-cluster coordinating residue (Gly279). In *Met*CODH (ACS not bound), helix αA shifts to block the gas channel, with Phe601 also swinging in to block the channel.

In our structure of *Met*CODH/ACS, the channel between the C-cluster and A-cluster is open and resembles the open channel near the A-cluster in *Moor*CODH/ACS (Fig. 5). In both cases, the channel runs between two helices (labeled αA and αB in *SI Appendix*, Figs. S2*B* and S3*A*) and terminates in the CO-binding alcove (Fig. 5 and *SI Appendix*, Fig. S18). In the structures of *Met*CODH without ACS bound (Fig. 1), this CO channel is closed. The structural basis for channel closing appears similar to that previously observed for *Moor*CODH/ACS (31). In both cases, helix αA shifts toward helix αB to close the gas channel and block CO release. Instead of Val507(*Met*)/Val149(*Moor*), Leu504(*Met*)/Ile146(*Moor*), and Phe601(*Met*)/Phe229(*Moor*) creating an alcove for bound-CO (Fig. 4 and *SI Appendix*, Fig. S15), these residues now create a choke point in the CO channel (Fig. 5 and *SI Appendix*, Figs. S18 and S19). Additionally, helix αA residue Arg500(*Met*)/Arg142(*Moor*) has repositioned so that it no longer hydrogen bonds to the A-cluster coordinating Gly279(*Met*)/Gly596(*Moor*) (Fig. 5 and *SI Appendix*, Figs. S18 and S19). This type of protein motion in CODH is quite interesting, as conformational motion is not precedented in CODHs. Typically, ACS is known to be the highly dynamic protein in the CODH/ACS system, but here, Rossmann fold 2 of CODH takes the place of A1 of ACS, and as such also takes on a similar conformational change. Prior to this work, relatively little was known about gas channels in methanogenic ACDS complexes (28, 47). Now we see that methanogenic CODH/ACSs do have a channel connecting their C- and A-clusters and that the channel can open and close using a similar mechanism to that observed in acetogenic CODH/ACSs. In both cases, channel closing prevents CO release during the time that ACS domains reposition to interact with CFeSP for methyl transfer.

## Discussion

There are at least six known pathways for CO_2_ fixation (48, 49). The majority involve adding a molecule of CO_2_ to an already formed carbon chain. The chemistry catalyzed at the A-cluster is distinctive in that it creates a two-carbon chain from two one-carbon units. This carbon fixation ability is one of several reasons that A-cluster chemistry is believed to be ancient, perhaps predating protein synthesis with slurries of nickel, iron, and sulfur creating carbon chains (1). When the first structure of an acetogenic CODH/ACS was reported two decades ago (25), the role(s) of the protein in acetyl-CoA synthesis emerged. Although the Ni–Fe–S cluster is the catalyst that joins CO with CH_3_, the protein controls CO access to the A-cluster via a gas channel that can open and close, and the protein controls CH_3_ group access via ACS domains that open and close. The protein can also sequester and stabilize A-cluster reaction intermediates and tune redox potentials. Notably, the acetogenic CODH/ACS structure showed that it was the nonconserved A1 of ACS that seemed most critical for these functions, raising questions as to whether the CODH/ACS component of ACDS would function in an entirely different way and whether the role of protein in A-cluster chemistry is in fact conserved.

Twenty years after the first structure of a CODH/ACS, we now know that the role of the protein in the A-cluster-catalyzed acetyl-CoA synthesis is functionally conserved and that this conservation includes the housing of gas channels. As observed previously in acetogens (46), we find that the methanogenic protein also has a channel between its C- and A-clusters that plays a role of CO gatekeeper. Interestingly, the channel is present in monofunctional *Dv*CODH but is not the one that is used by either acetogenic *Moor* or *Ca*CODH (26, 45, 46). Monofunctional CODHs have three pathways that have been identified by xenon studies that extend from the C-cluster to the surface of CODH: channel 1, 2A, and 2B (Fig. 4) (45). We now have structures of three different CODH/ACSs, two from acetogens and one from a methanogen, as reported in this work. In these complexes, each CODH binds ACS in a different way, and each CODH/ACS complex utilizes a different gas channel as a result. Previous studies have described how amino acid substitutions in CODH/ACSs are likely to close off channels to direct CO to that enzyme’s A-cluster (45). The fact that we now see 3 of 3 xenon-identified routes employed in a CODH/ACS raises the question as to whether we have now observed all the various ways in which an ACS can bind to a CODH.

Notably, the channel in methanogenic CODH/ACS is much shorter than it is in either of the structurally characterized acetogenic enzyme complexes. The longer channel in the acetogenic systems is created by the presence of the A1 domain, which extends the gas channel into the ACS subunit. A longer channel could provide a storage chamber or “waiting room” for CO in the acetyl-CoA synthetic direction, in which a slow protein conformational change at the A-cluster occurs in between each CO consumption step. However, there is no experimental evidence that multiple CO molecules reside in the gas channel under turnover conditions. The observed difference in gas channel lengths can still be rationalized if only one CO molecule exists in the channel at a time. There are two options for a CO molecule in the gas channel: CO can either be consumed by oxidation at the C-cluster or consumed by acetyl-CoA synthesis at the A-cluster. The K_M_ for CO oxidation at the C-cluster is higher than the K_M_ for acetyl-CoA synthesis at the A-cluster. This K_M_ difference favors CO oxidation when CO concentrations are high and favors acetyl-CoA synthesis when CO concentrations are low. One CO molecule in a short channel has an effective concentration that is higher, which would favor CO oxidation. One CO molecule in a large channel has a lower effective concentration, which would favor acetyl-CoA synthesis. Thus, the addition of the A1 domain to acetogenic CODH/ACSs could have been an adaptation to lower the effective concentration of CO, thereby favoring acetyl-CoA synthesis, and possibly avoiding a futile cycle in which the C-cluster consumes the CO that it produced.

It is interesting to speculate on the evolutionary history of the A1 domain. A comprehensive phylogenomic study of CODH/ACSs that utilized over 6,400 archaeal and bacterial genomes, supports the presence of a CODH/ACS in the LUCA, but the precise nature of that CODH/ACS could not be predicted (4). The close structural homology between the Rossmann 2 domain of CODH and the A1 domain of bacterial ACSs suggests a gene duplication event (Fig. 3). It is possible that this gene duplication happened before archaea and bacteria split, and that the A1 domain was lost to archaeal ACSs early on, explaining the lack of this domain in all modern archaeal ACSs. Alternatively, the LUCA CODH/ACS might not have had an A1 domain. The A1 domain could have been added later via gene duplication of CODH Rossmann domain 2, perhaps providing a selective advantage to CODH/ACSs that utilize CO_2_ as an electron sink, forming CO en route to synthesizing acetyl-CoA. Such a selective advantage would explain why this domain is found only in acetogenic bacteria but not in methanogenic archaea.

Unexpectedly, we find that acetogenic and methanogenic CODH/ACSs share the same mechanism of closing the CO gas channel, i.e., movement of helix αA toward helix αB (Fig. 5). In acetogenic CODH/ACS, it is helix αA of ACS domain A1 that moves, and in methanogenic CODH/ACS, it is helix αA of CODH Rossmann 2 that moves. Despite this difference, the mechanism appears conserved. This conservation of the channel plugging mechanism suggests a functional importance for preventing escape of CO. For acetyl-CoA synthesis, the generation of CO at the C-cluster comes at a cost of two low-potential electrons. Retaining CO in the channel of an acetogenic CODH/ACS ensures that low-potential reducing equivalents are not wasted. The low-potential electrons generated from CO oxidation are also crucial for acetoclastic methanogens, as they are used to drive reduction of the acetyl-CoA-derived methyl group to methane (11). Escape of CO from the channel into the cell could additionally lead to unwanted binding of CO to other metal centers or release of CO from the cell. For animals harboring acetogens and methanogens in their intestinal tracts, release of CO could be toxic (50–52). Therefore, it would be disadvantageous to leave the gas channel open when ACS has rearranged to engage with CFeSP.

In addition to being a gatekeeper for CO, the protein directly facilitates the binding of CO, CH_3_, and/or COCH_3_ to the correct coordination site(s) on the A-cluster for catalysis, stabilizing bound states with the correct geometry for catalysis, and protecting such states from unwanted side reactions. In acetogens, A1 of ACS is responsible for these roles, and in methanogens, it is Rossmann 2 of CODH. The conservation of amino acids involved in these interactions is remarkable (Fig. 3). As observed for acetogenic enzymes, we find that a CO channel directs CO molecules to bind to the proximal Ni of the A-cluster (Ni_p_), and that residues of an alcove ensure that Ni_p_-CO adopts tetrahedral geometry (29). This alcove, composed of Leu504, Val507, and Phe601 from *Met*CODH and Phe195 from *Met*ACS, also appears large enough for an acetyl moiety to fit while maintaining tetrahedral geometry of Ni_p_. It was previously proposed that sequestration of the acetyl-bound A-cluster must be important to prevent hydrolysis, which would form acetate rather than the valuable metabolic intermediate acetyl-CoA (46). A protein alcove above Ni_p_ could thus have multiple functions. The protein also must play a critical role in ensuring the correct electron distribution in the A-cluster for the various steps of catalysis, and we find conserved amino acid residues support similar interactions in both acetogens and methanogens. These interactions include an Arg residue (Arg500 in *Met*) that hydrogen bonds to A-cluster-coordinating residue Gly279.

**Fig. 6.**
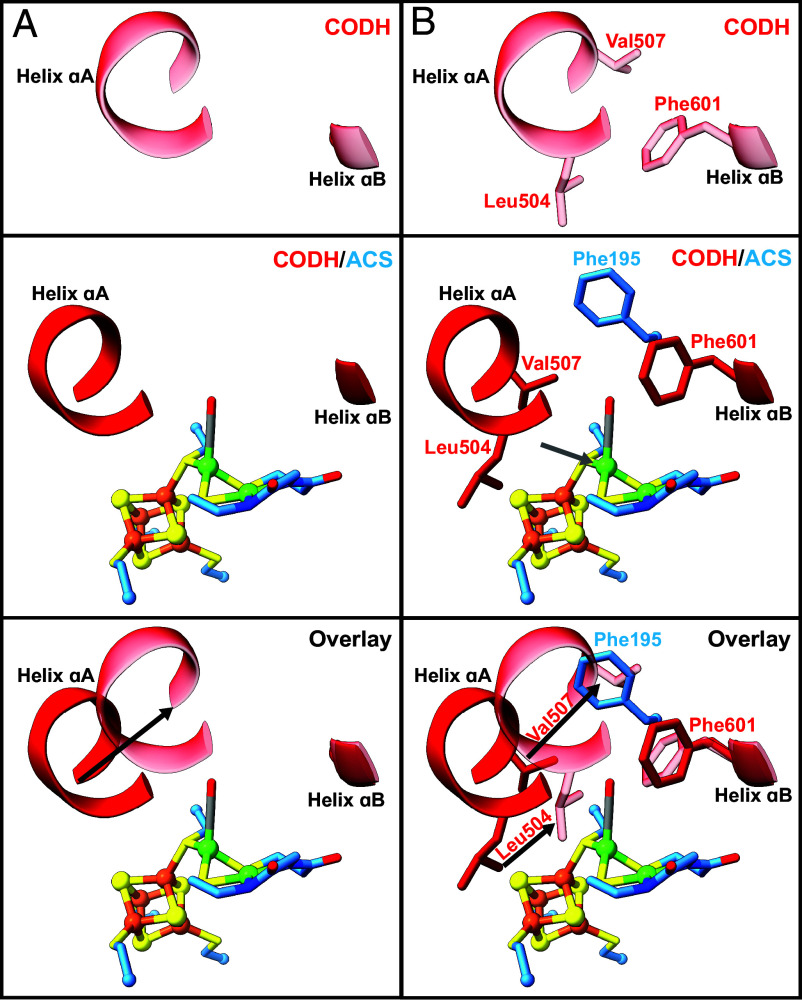
Conformational change in *Met*CODH helix αA and alcove residues should facilitate ACS departure from CODH. (*A*) Secondary structural movements in *Met*CODH. *Top*: Structure of *Met*CODH alone (transparent red). *Middle*: Structure of *Met*CODH (red)/ACS (blue). *Bottom*: Overlay of *Met*CODH (red)/ACS (blue) and *Met*CODH alone (transparent red) structures, showing a large shift in helix αA. (*B*) Residue movements in *Met*CODH. *Top*: Structure of *Me*tCODH alone, showing positions of alcove residues Leu504, Val507, and Phe601. *Middle*: Structure of *Met*CODH (red)/ACS (blue), showing the position of *Met*CODH alcove residues Leu504, Val507, Phe601, and *Met*ACS residue Phe195. *Bottom*: Overlay of *Met*CODH (red)/ACS (blue) and M*et*CODH alone (transparent red) structures, showing movement of Leu504 toward Ni_p_ and of Val507 toward Phe195. Black arrows are used to highlight helix and residue movements. The gray arrow in panel *B* indicates the putative methylation site on Ni_p_. For clarity, only residues discussed in the text are shown.

**Fig. 7.**
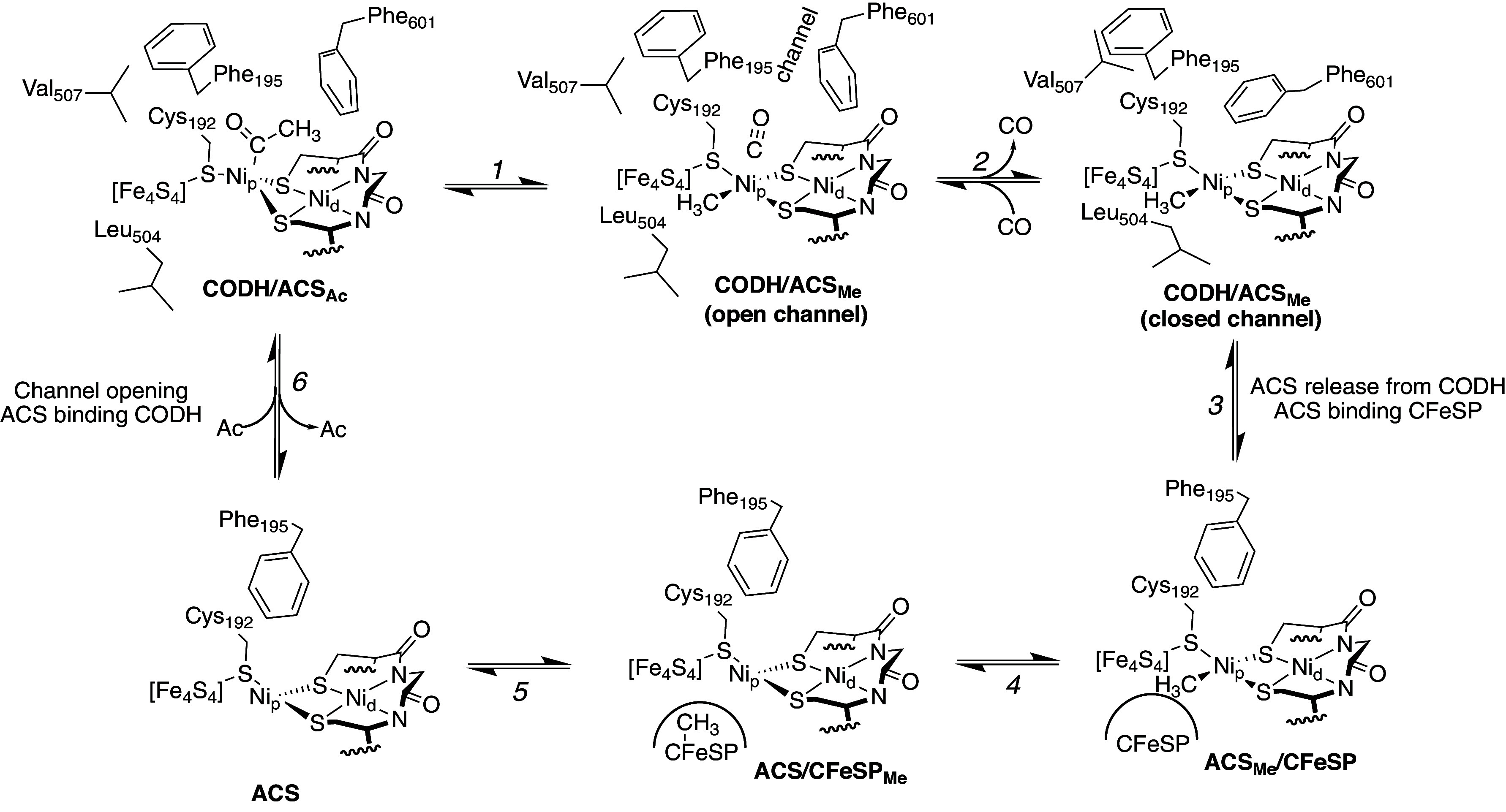
Proposed protein conformational changes and resulting effects on the A-cluster environment in the mechanism of acetyl-CoA cleavage in ACDS. Steps are labeled 1 to 6. The proposed CODH/ACS_Ac_ state is based on the geometry and residue positions of the CO-bound structure (this work, see Fig. 6 *B*, *Middle*). Modeling suggests that an acetyl moiety can fit in the alcove but cannot fit in the predicted methyl coordination site due to the close positioning of Leu504. The geometry of the A-cluster shown for all ACS_Me_ states is based on X-ray absorption spectroscopy on *Moor*ACS (53). Residue positioning for CODH/ACS_Me_ open-channel and closed-channel states is based on the observed residue positions (this work, Fig. 6*B*). The positioning of Phe195 when *Met*ACS is not bound to *Met*CODH is based on the position of the analogous Phe512 in the closed-channel ACS structure of *Moor*CODH/ACS (Fig. 5 *A*, *Right*) (31) and also on the loss of protection from CO inhibition when Phe195 of *Met*ACS is substituted with Ala (43).

Another key role for the protein in A-cluster chemistry is to alternatively position the A-cluster for carbonylation by CODH and for methylation by CFeSP. No structure of ACS bound to CFeSP exists, but the structures of CODH/ACSs, including the one reported herein, indicate that domains must move for the A-cluster to be exposed and bind CFeSP. With the available structural and biochemical data, we can think about the conformational rearrangements that must occur during acetyl-CoA cleavage in a methanogenic ACDS complex (Figs. 6 and 7). Starting with acetylation of the A-cluster, an acetyl-bound Ni_p_ would be expected to disassemble into CO and a CH_3_ with both moieties migrating to separate coordination sites on Ni_p_ (Fig. 7, step 1). The CO should dissociate from Ni_p_ via an open CO channel (Fig. 7, CODH/ACS_Me_ open channel) and the resulting methyl-Ni_p_ is expected to adopt square planar geometry based on X-ray absorption spectroscopy on *Moor*ACS (53). Adoption of square planar geometry positions the methyl group out of the alcove. With the alcove empty, helix αA is free to move toward helix αB. The observed ~5-Å movement of helix αA closes the channel but also should destabilize the ACS:CODH interface with CODH residue Val507 moving into the location occupied by alcove residue Phe195 of ACS and Leu504 moving close to the expected binding site for the methyl moiety on Ni_p_ of the A-cluster (Fig. 6). A closed-channel state should therefore have weaker affinity for the methylated-A-cluster than the open-channel state, shifting the equilibrium toward ACS dissociation (Fig. 7, step 3). The correlation between the liganded state and geometry of Ni_p_ with the state of the channel (open or closed) is also found in previous structural data; when Ni_p_ adopts a tetrahedral geometry, the gas channel is open, whereas when Ni_p_ adopts a square planar geometry, the gas channel is closed (*SI Appendix*, Table S2) (25, 29, 31, 54–56). Notably, interface analysis via PDBe PISA (57) shows that the number of contacts between *Met*CODH and *Met*ACS is modest (*SI Appendix*, Tables S3–S5), meaning that the binding interaction should be very sensitive to changes in protein structure and/or ligated state of the A-cluster. After ACS dissociates from CODH, ACS must associate with CFeSP for methyl transfer, forming the methylcorrinoid species (Fig. 7, step 4) (58, 59). The ACS/CFeSP complex then dissociates (Fig. 7, step 5) and ACS can reassociate with CODH (Fig. 7, step 6).

In the proposed reaction scheme for acetyl-CoA synthesis by *Met*CODH/ACS depicted in Fig. 7, methylation precedes carbonylation. The order of the reaction in *Moor*CODH/ACS has been debated in the literature (60) with arguments made for a random-order mechanism with either carbonylation first or methylation first (61), and also for an ordered mechanism with methylation first (62). A consensus has not yet been reached. In contrast, *Met*CODH/ACS has only been proposed to operate by a methylation-first mechanism based on extensive biochemical analyses (43, 47). In particular, the CO-bound A-cluster of *Met*ACS has been shown to be an inhibited form of the A-cluster that cannot be methylated (43). This inhibition is reversible, and the A-cluster can lose CO and then be methylated (43). The structural data presented here provide a molecular explanation for both of these experimental observations. *Met*ACS must dissociate from CODH to be methylated by CFeSP, and carbonylation of the A-cluster should decrease the tendency for CODH/ACS to dissociate because of the packing interactions made between CO and alcove residues thereby inhibiting methylation. Also, the structure suggests why CO inhibition is reversible: a CO molecule residing in the alcove can diffuse back into the gas channel, which would lead to helix αA movement, prompting ACS departure. Once dissociated from CODH, *Met*ACS would be free to interact with CFeSP for methylation. By disfavoring *Met*ACS departure from CODH while the A-cluster is in the carbonylated state, the system protects against CO molecule loss that might occur while ACS is in transit.

To prevent formation of a CO-inhibited state, one can imagine that *Met*CODH/ACS has protein design features that limit the amount of carbonylation that can occur on an unmethylated A-cluster, and those features do appear to be present. As described above, Leu504 of helix αA is directly adjacent to the postulated methyl-coordination site on the A-cluster, and when the channel is closed, the distance would be unfavorably close (Fig. 6*B*). Such a close interaction would be expected to shift the conformational equilibrium of helix αA toward the open-channel conformation, allowing for A-cluster carbonylation of a methylated A-cluster. By contrast, in the absence of a methyl ligand, ACS should still be able to bind to *Met*CODH, but without shifting the position of helix αA, thereby maintaining a closed CO channel and limiting carbonylation and thus CO-inhibition of the A-cluster (43).

The structure obtained here of *Met*CODH/ACS likely relates to the previously described CO-inhibited state (43). It has not been possible to confirm the identity of the A-cluster ligand as CO via spectroscopy since we did not work with purified *Met*CODH/ACS but rather with purified ACDS that dissociated into multiple subcomplexes upon cryo-EM grid preparation (Fig. 1). Since ACDS was purified from the native organism while engaged in methanogenesis, the protein components likely represented a mixture of redox and liganded states. We hypothesize that when ACDS was isolated, some CO molecules remained in the channel. CO can be quickly consumed at the C-cluster, but only if the C-cluster is in the proper oxidation state (known as Cred1) (63). If the C-cluster oxidation state was improper, or if electron acceptors such as ferredoxin become unavailable (due to cellular disruption and dilution) this could result in residual, unconsumed CO within the gas channel. CO that remained in the channel would then have the option to return to bind at the A-cluster (after reassociation of ACS with CODH), and once bound, stabilize the CODH/ACS interaction due to favorable interactions in the CO-binding alcove, allowing us to visualize the complex on the EM grid. Although the increased stability afforded by a CO ligand in the alcove is substantial, it is unlikely large enough to allow for purification of the CODH/ACS subcomplexes by conventional methods. Accordingly, CODH/ACS particles were only visualized in our chameleon-plunged specimen, which is considered a more rapid and gentler vitrification method. Even in our chameleon-plunged specimen, most of the observed CODH particles did not have ACS bound. In short, determination of this methanogenic CODH/ACS structure would not have been possible without the “resolution revolution of cryo-EM” (64) or without the improved particle picking and sorting software that allows for structures to be obtained from heterogeneous protein samples (65–68) or without the development of gentler blot-free plunging methods (36, 37, 39).

In conclusion, these structural studies have provided a long-awaited snapshot of a methanogenic CODH/ACS complex. However, the structural work is not complete. We are still lacking visualization of the interaction between ACS and CFeSP from any organism, which would provide much needed insight into the methyl transfer step of ACS catalysis. We are also missing structural data on the C-terminal extension of *Met*ACS that is likely to play a role in the conformational rearrangements that ACS must make as it swings between CODH and CFeSP within the 2.2 MDa ACDS complex. Further study of the ACDS complex will be crucial for understanding the unique aspects of this system. Although the structures of acetogenic and methanogenic systems for acetyl-CoA synthesis and cleavage show marked differences, our work demonstrates that these systems have more in common than previously appreciated, including a conserved CO-binding alcove and channel gating mechanisms. These structural insights are likely to promote renewed discussion of the mechanism of the A-cluster of ACS, bringing us closer to a unified understanding of how nature makes acetyl-CoA from CO_2_-derived one-carbon units, and how it breaks acetyl-CoA into two one-carbon units en route to the production of greenhouse gases CO_2_ and methane. Although this chemistry is ancient, it is no less relevant today as we seek novel ways to reduce the levels of greenhouse gases in our environment.

## Materials and Methods

### Growth of *M. thermophila*.

The method for growth of *Methanosarcina barkeri* on acetate (69) was modified for large-scale growth of *M. thermophila*, as described in detail (70). Briefly, *M. thermophila* strain TM-1 was grown at 50 °C in a minimal salts medium containing 2.5 g/L sodium acetate and 2.5 g/L potassium acetate trihydrate, supplemented with 0.15 g/L yeast extract. Anaerobic medium was reduced with 0.175 g/L cysteine-HCl monohydrate and 0.075 g/L sodium sulfide nonahydrate. Actively growing starter cultures were used for 1:10 inoculation of 20 L batch cultures. Glacial acetic acid was added during growth to maintain pH between 6.4 and 6.8. After gas production and acetic acid consumption reached a maximum, constant rate (~5 to 7 L gas evolved per hour) 50 to 70 h after inoculation, the cultures were chilled and then harvested anaerobically using a flow-through Sharples type centrifuge. Yield was 60 to 80 g cell paste per 20 L batch, which was frozen in liquid N_2_ and stored in the vapor phase of a liquid N_2_ storage unit.

### Purification of the ACDS Complex.

Preparation of soluble extracts from *M. thermophila* and isolation of the ACDS complex followed the same general approach as described for *M. barkeri* (11) except that the procedure was scaled down for use inside a Coy type anaerobic chamber. Briefly, frozen *M. thermophila* cell paste, 10 to 12 g, was thawed with addition of 30 mL of anaerobic Buffer A (50 mM MOPS-Na and 100 mM Na_2_SO_4_, pH 7.2) and disrupted by a single pass through a chilled 40-mL French press cell (1-inch diameter piston) at 18,000 to 20,000 psi. Following addition of DNase I to reduce the viscosity, the extract was centrifuged at ~48,000×*g* for 22 min at 0 to 4 °C, and the supernatant was collected. Additional discussion of the extraction procedure, including the techniques used to maintain anaerobic conditions throughout, can be found in ref. 70.

For purification of ACDS, the extract supernatant was applied to a column of Sepharose CL-6B (95 × 2.6 cm diameter) equilibrated in anaerobic Buffer A at a rate of 0.75 mL/min. Fractions were collected at 10 min intervals and assayed for protein (Bradford reagent, BioRad) (71) and CO dehydrogenase activity (in reaction mixtures containing 10 mM methylviologen and 0.1 M Tris-HCl, pH 8.0, saturated with 100% CO at 25 °C). The ACDS complex eluted as a brown, high-molecular-weight protein peak that emerged from the column after a series of gray-colored turbid fractions, marking the void volume, and before the peak of methyl-coenzyme M reductase, identified as a green-colored zone which faded to yellow within several hours after collection. The ACDS elution position is consistent with a 2 MDa complex, and the elution profile is similar to what was found for ACDS from *M. barkeri* in previous work (11). Analytical size exclusion chromatography using a Superose 6 HR column showed a very large complex (also consistent with the expected ~2 MDa size), and most notably the near absence of smaller particles of α_2_ε_2_ CODH (220 kDa, which elutes at the 26 min position)—see *SI Appendix*, Fig. S20. The high molecular weight ACDS peak exhibited approximately constant CODH-specific activity, and 4 to 7 (usually 5) high-molecular fractions with the highest activity were pooled. A 50-mL Amicon stirred cell with YM30 ultrafiltration membrane was then used at ~10 psi N_2_ overpressure to concentrate the ACDS pool to about 8 to 10 mL. Diafiltration was then performed to reduce the salt concentration prior to freezing, by adding 10 mL of H_2_O to the concentrate and reconcentration, which was done twice, ending at a final volume of ~5 mL. The ACDS concentrate was drip-frozen in liquid N_2_ and stored in the vapor phase of a liquid N_2_ storage system. Addition of ~1,000 U Benzonase nuclease (Sigma) and 1 mM MgCl_2_ to the pool prior to concentration resulted in preparations with noticeably lower absorbance at 260 nm. The final A_280_/A_400_ ratio was typically in the range of 4.8 to 5.0. Under the assay conditions described, ACDS exhibited a CODH-specific activity of 34.9 ± 2.3 U/mg (n = 6). The preparations also exhibit corrinoid methyltransfer activity using methylcobinamide as a donor and tetrahydrosarcinapterin (H_4_SPt) as acceptor. CO, CO_2_, and CoA exchange activities were characterized previously as well on similarly prepared ACDS sample to confirm full ACDS activity under these preparation conditions.

### Cryogenic Electron Microscopy Grid Preparation.

To prepare blot-plunged cryo-EM samples, an aliquot of purified ACDS was thawed inside of a Coy anaerobic chamber (N_2_/H_2_ atmosphere) and diluted to a concentration of 1 mg/mL in 25 mM MOPS and 25 mM Na_2_SO_4_, pH 7.2. The protein sample was supplemented with 0.1% glutaraldehyde. Quantifoil 1.2 μm hole, 1.3 μm spacing Cu 300 mesh holey-carbon grids (EMS) were glow-discharged for 60 s at −15 mA using a PELCO easiGlow system (Ted Pella). Grids were brought into the anaerobic chamber directly after glow discharging. Grids were loaded into the blotting chamber of a Cryoplunge 3 device (Cp3, Gatan), which was maintained at >80% humidity, and allowed to humidify for ~30 s before 3 μL of sample was applied to the carbon-coated side of the grid. The sample was left for ~30 s before blotting for 4 s, with blot settings of −1.0 mm on the sample-facing blotter and −0.5 mm on the back-facing blotter, and plunged in liquid ethane. Grids were transferred to buttons under liquid nitrogen, removed from the anaerobic chamber, and stored under liquid nitrogen.

To prepare chameleon-plunged cryo-EM sample, an aliquot of purified ACDS was thawed inside of a Coy anaerobic chamber and diluted to a concentration of 4 mg/mL in 25 mM MOPS and 25 mM Na_2_SO_4_, pH 7.2. The protein was flash-frozen in liquid nitrogen, transported to the chameleon (SPT Labtech) instrument frozen under liquid nitrogen, thawed, loaded into the chameleon sample vial, and immediately aspirated into the chameleon dispenser. Self-wicking 1.2 μm hole, 0.8 μm spacing holey-carbon grids (SPT Labtech) were glow-discharged for 35 s at −12 mA using the chameleon on-board glow discharger (SPT Labtech). The cryo-EM specimen was frozen using a dispense-to-plunge time of 150 ms (humidity 82%) and stored under liquid nitrogen.

### Cryo-EM Data Collection.

Samples were screened at the Cryo-EM facility in MIT.nano at the Massachusetts Institute of Technology (MIT) on a FEI Talos Arctica G2 Cryo 200 kV transmission electron microscope equipped with a Falcon 3EC camera. All data were collected at the Cryo-EM facility in MIT.nano at MIT on a Titan Krios G3i 300 kV Cryo transmission electron microscope equipped with a K3 Gatan Direct Detector using Thermo Fisher Scientific EPU software. For the specimen that was plunged on the Cryoplunge 3, a dataset was collected at a magnification of 105,000x in superresolution mode and binned by 2 during collection, resulting in a pixel size of 0.832 Å. In total, 14,078 movies were collected with 21 frames, 54.53 e^−^/Å^2^ total exposure, and a defocus range of −0.8 to −2.1 μm (*SI Appendix*, Table S1). For the specimen that was plunged on the chameleon, a dataset was collected at a magnification of 130,000× in superresolution mode and binned by 2 during collection, resulting in a pixel size of 0.654 Å. In total, 13,892 movies were collected with 40 frames, 47.1 e^−^/Å^2^ total exposure, and a defocus range of 0.0 to −1.75 μm (*SI Appendix*, Table S1).

### Cryo-EM Data Processing.

Cryo-EM data processing was carried out using a combination of cryoSPARC v3.3.2 (65), pyem v0.5 (72), RELION v4.0 (66), and cryoDRGN v0.3.4 (67, 68). For the dataset collected on the Cp3-plunged grid, the data processing workflow is summarized in *SI Appendix*, Fig. S5. For the dataset collected on the chameleon-plunged grid, the data processing workflow is summarized in *SI Appendix*, Figs. S7–S9. See *SI Appendix* for details.

### Model Building and Coordinate Refinement.

A starting model of CODH/ACS hexamer was generated using the COSMIC2 implementation (73) of AlphaFold multimer (74), which gave correct overall shape at low resolution (maps of 5 Å resolution or lower), but upon experimental visualization of secondary structure via EM density, it was evident that the conformation of CODH/ACS differed significantly from the AlphaFold prediction. CODH/ACS AlphaFold prediction was split into components: CODH α_2_ε_2_, ACS β residues 1 to 183, ACS β residues 184 to 397. Residues 398 to 472 of ACS β were excluded, as there was no EM density present in any map consistent with this C-terminal region of ACS. CODH α_2_ε_2_ was docked into all four maps (CODH tetramer Cp3-plunged, CODH tetramer chameleon-plunged, CODH/ACS pentamer, CODH/ACS hexamer) in ChimeraX (75). ACS β components were docked into CODH/ACS pentamer and CODH/ACS hexamer maps in ChimeraX. Each model was subjected to manual refinements in coot (76). Appropriate cofactors (Fe–S and Ni–Fe–S clusters) were added in coot. At the proximal nickel of the A-cluster (in the pentamer and hexamer), extra density was observed consistent with another ligand in addition to the two thiol (Cys278 and Cys280) and one sulfide ligands. Extra density was consistent with a CO ligand, which was modeled in coot. Models were iteratively real-space refined in Phenix (77) using noncrystallographic symmetry (NCS) restraints for the tetramer and hexamer. Custom parameter files were used to restrain the metallocluster geometries during refinement. In final stages of model building, C-terminal density of the β subunit was reexamined and the model was extended by a few residues where possible (*SI Appendix*, Table S1). Some of the software used was packaged by SBGrid (78). Data collection, refinement, and validation are presented in *SI Appendix*, Table S1.

### Computational Channel Prediction.

Internal channel prediction was performed by MOLEonline (79) using default cavity parameters including a probe radius of 5 Å (upper bound of channel radius) and an interior threshold of 1.1 Å (lower bound of channel radius).

## Supplementary Material

Appendix 01 (PDF)

## Data Availability

Coordinates and EM data have been deposited in the protein data bank (PDBID 9C0Q (80), 9C0R (81), 9C0S (82), and 9C0T (83), the electron microscopy data bank (EMDB) (EMD-45089 (84), EMD-45090 (85), EMD-45091 (86), and EMD-45092 (87)) and the electron microscopy public image archive (EMPIAR) EMPIAR-12108 (88) and EMPIAR-12124 (89). All other data are included in the manuscript and/or *SI Appendix*.
