# Supplementary material for: Capturing a methanogenic carbon monoxide dehydrogenase/acetyl-CoA synthase complex via cryogenic electron microscopy
Source: Proc Natl Acad Sci U S A. 2024 Oct 3;121(41):e2410995121. doi: 10.1073/pnas.2410995121 (PMC11474084; doi:10.1073/pnas.2410995121)
Supplement: Supplementary file 1 — Appendix 01 (PDF) [file pnas.2410995121.sapp.pdf]

Supporting Information for

Capturing a methanogenic carbon monoxide dehydrogenase/acetyl-CoA synthase complex via cryogenic electron microscopy.

Alison Biester, David A. Grahame, Catherine L. Drennan*

*Corresponding author: Catherine L. Drennan
Email: cdrennan@mit.edu

This PDF file includes:

Supplementary methods
Figures S1 to S20
Table S1 to S5
SI References

Supplementary methods

Cryogenic electron microscopy data processing

Cryo-EM data processing was carried out using a combination of cryoSPARC v3.3.2,[1] pyem v0.5,[2] RELION v4.0,[3] and cryoDRGN v0.3.4.[4-5] For the dataset collected on the Cp3-plunged grid, the data processing workflow is summarized in **Figure S5**. First, the 14,078 movies were subjected to patch motion correction and patch CTF estimation in cryoSPARC. Exposures were manually curated based on CTF fit resolution using a cutoff of 7.83 Å, leaving 13,270 remaining micrographs. The 13,270 remaining micrographs were divided into 8 subsets, one of which was used for blob picking (elliptical blob) with a minimum particle diameter of 75 Å and a maximum particle diameter of 150 Å. Particles were extracted with a box size of 288 px and 2D classified to generate templates. Templates were selected from 2D classes. Micrographs were then divided into 6 subsets (to increase processing speed) each of which was subjected to template picking with a particle diameter of 150 Å. In total, 852,015 particles were extracted from micrographs with a box size of 288 px. Particles were 2D classified into fifty classes. Classes consistent with the shape of CODH were selected, giving 561,072 particles after 2D classification. Selected particles were used to generate *ab initio* models with 2 classes and the particles from the best class were used to move forward (347,086 particles). The pyem v0.5 software csparc2star was used to generate a RELION star file from the cryoSPARC *ab initio* particle stack and optics groups were added to the star file. In RELION, particles were subjected 3D classification with six classes, from which the best class was selected, containing 141,277 particles. Those particles were used to generate a new initial model with C2 symmetry, which was subjected to 3D refinement. Another 3D classification with four classes was run with C2 symmetry applied. The best class was selected, resulting in a final selection of 99,894 particles. Particles were downsampled to a pixel size of 1.17 Å and used to generate a 3D initial model with C2 symmetry, which was then subjected to 3D refinement and CTF refinement. Particles then went through Bayesian polishing and an additional round of CTF refinement prior to the final round of 3D refinement and postprocessing, which gave a global resolution of 3.3 Å (**Table S1**). Fourier shell correlation (FSC) plot, angular distribution plot, 3D FSC plot generated using the 3D FSC web server,[6] and local resolution are presented in **Figure S6**.

For the dataset collected on the chameleon-plunged grid, the data processing workflow is summarized in **Figures S7-S9**. First, 13,892 movies were subjected to patch motion correction and patch CTF estimation in cryoSPARC. Exposures were manually curated based on CTF fit resolution using a cutoff of 7.00 Å, leaving 13,067 remaining micrographs. In the micrographs, it was evident that CODH particles were present, but the presence of ACS bound was not obvious. The 13,067 remaining micrographs were divided into 9 subsets, two of which were used for blob picking (elliptical blob) with a minimum particle diameter of 75 Å and a maximum particle diameter of 175 Å. Particles were extracted with a box size of 400 px and 2D classified to generate templates. Upon generation of templates, it became evident that some particles had one ACS bound. Therefore, the templates that resembled CODH were selected and separately, templates resembled CODH/ACS were selected. For CODH template picking, a particle diameter of 150 Å was used, whereas for CODH/ACS

template picking, a particle diameter of 175 Å was used. All particles were extracted with a box size of 400 px (836,578 particles from CODH template picks and 754,444 particles from CODH/ACS template picks, **Figure S7**). At the 2D classification stage, 836,578 CODH template picks were 2D classified into fifty classes, at which point it became clear that the picks from the CODH template included both CODH and CODH/ACS. Therefore, all classes that resembled CODH or CODH/ACS were selected, giving 343,693 particles. The 754,444 CODH/ACS template picks were also 2D classified into fifty classes and the classes that resembled CODH or CODH/ACS were selected, giving 220,526 particles. Each set of selected particles was used to make *ab initio* models with three classes. In each *ab initio* model job, there was one class that had mainly intact particles, with a mixture of CODH and CODH/ACS, whereas the other two classes contained junk and/or partially denatured particles (insufficient density for even the CODH tetramer). Therefore, from each *ab initio* model job, the best of the three classes was selected and subjected to removal of duplicate particles. The removal of duplicate particles was necessary because two rounds of template picking were completed, leaving the possibility that some particles were picked twice and included in both *ab initio* models. After the removal of duplicate particles, 197,578 particles remained. The remaining 197,578 particles were used to generate an *ab initio* model (one class).

Once it became clear that the template picker was unable to distinguish between CODH and CODH/ACS, in a parallel strategy, the 836,578 particles from CODH template picks and 754,444 particles from CODH/ACS template picks were subjected to duplicate removal, yielding 1,124,872 particles remaining. Those 1,124,872 particles were classified into fifty 2D classes from which classes were selected that resembled CODH and/or CODH/ACS, giving 561,690 selected particles. To best utilize the results from both strategies, the remaining 197,578 particles selected from the ab initio models were combined with the 561,690 particles selected from 2D classification using a duplicate removal job, giving 583,270 particles remaining. Those remaining particles were used to generate an *ab initio* model with two classes. One class appeared to contain mostly denatured particles whereas the second class contained mostly intact complexes of CODH or CODH/ACS. The best class from the *ab initio* model job was put through the pyem v0.5 software csparc2star to generate a RELION star file and optics groups were added to the star file (392,344 particles). Particles were re-extracted in RELION and 3D classified into six classes, using a mask diameter of 200 Å, which was used as the mask diameter throughout refinements and classifications. The best four classes were selected, leaving 342,404 particles, which were subjected to 3D refinement yielding a map of 4.3 Å resolution that contained a mixture of CODH and CODH/ACS (bottom of **Figure S7**).

During the RELION 3D classifications, it became evident that there were two different CODH/ACS species: a CODH/ACS pentamer containing the $\alpha_2\varepsilon_2$ CODH tetramer and one β ACS monomer and a CODH/ACS hexamer containing the $\alpha_2\varepsilon_2$ CODH tetramer and two β ACS monomers. Therefore, the next step was to separate the CODH tetramer, CODH/ACS pentamer, and CODH/ACS hexamer into three different selections of particles from the total 342,404 particles.

With a pool of particles containing the desired species in hand, next, the species were separated using a combination of RELION 3D classification and cryoDRGN with

downstream implementation of Model-based Analysis of Volume Ensembles (MAVEn) (**Figure S8**). The outputs of these strategies were combined using the duplicate removal tool in cryoSPARC. In the RELION workflow (left side of **Figure S8**), the 342,404 remaining particles were subjected to 3D classification into six classes. Of the six classes, two appeared to have no density for ACS, containing CODH only, whereas four classes had some density for at least one ACS. The four classes that had some density for at least one ACS were selected (184,188 particles) and reclassified into six 3D classes. From the six classes, the best class containing CODH and some density for ACS was selected (105,864 particles). Those 105,864 particles were again 3D classified into six classes. From those six classes, one class containing a mixture of CODH/ACS pentamer and hexamer was selected (39,237 particles). Those particles were then classified again into six classes. From those six classes, one class containing 25,256 pentamer particles was selected (bottom left of **Figure S8**). This class was selected due its apparent compositional homogeneity; the class had similar strength of EM density for the CODH subunit and one ACS subunit. Also, one class containing 11,875 hexamer particles was selected (bottom left of **Figure S8**). Again, this class was selected due its apparent compositional homogeneity; the class had similar strength of EM density for the CODH subunit and both ACS subunits. From the classification of all 342,404 remaining particles, the other two classes containing density for only CODH were separately selected, used to make a new initial model with C2 symmetry, refined, and classified into four classes, from which three were selected (153,285 particles). From the classification of 105,864 particles, one class (44,445 particles) was found to have very little density for ACS and was therefore selected separately to be combined with the CODH particle pool. The combined 153,285 particles and 44,445 particles from two separate classifications were combined and reclassified into six classes. The best class with 136,062 CODH particles was selected (bottom left of **Figure S8**).

The preliminary reconstructions from RELION 3D classification were sufficient to dock in all subunits: $\alpha_2\varepsilon_2$ to the CODH tetramer map, $(\alpha_2\varepsilon_2)\beta$ to the CODH/ACS pentamer map, and $(\alpha_2\varepsilon_2)\beta_2$ to the CODH/ACS hexamer map (described in Model building and coordinate refinement section of main text). With docking models and a consensus refinement at 4.3 Å resolution, we were then able to utilize cryoDRGN and MAVEn.

In the cryoDRGN workflow (right side of **Figure S8**), poses and CTF parameters from the 3D refinement were converted to cryoDRGN format. The dataset was downsampled to a box size of 128 pixels and split into 50,000 particle substacks. The first 10,000 particles were backprojected to verify that all data were parsed correctly. A cryoDRGN network was trained on the 128 pixel downsampled particles. After the training, it was confirmed that the network had converged. An automated analysis was performed on the trained cryoDRGN model at epoch 49 using the cryoDRGN analyze tool. Volumes were generated at 100 *k*-means clusters centers. The volumes were examined in ChimeraX[7] to identify “junk clusters,” which were then excluded from further analysis using the interactive Jupyter notebook generated from the cryoDRGN analyze tool, leaving 284,105 particles after filtering. A RELION star file was generated corresponding to the filtered indices and a 2D classification was run in RELION to verify that the filtering was satisfactory. Next, the particles were downsampled to a box size of 256 pixels and split into 50,000 particle substacks, and the first 10,000 particles were

backprojected to verify that all data were parsed correctly. A cryoDRGN model was trained on the 256 pixel downsampled particles. After the training, it was confirmed that the network had converged. An automated analysis was performed on the trained cryoDRGN model at epoch 49 using the cryoDRGN analyze tool. Volumes were generated at 500 *k*-means clusters centers. Then, to begin the occupancy analysis via the MAVEn software, an atomic model of the CODH/ACS hexamer, $(\alpha_2\varepsilon_2)\beta_2$ was aligned to a cryoDRGN volume that contained density for all subunits. The atomic model had six chains defined: two α chains, two ε chains, and two β chains. MAVEn occupancy analysis scripts were used to generate mrc files for each chain and masks for each mrc file. Then, the occupancy analysis scripts were used to calculate reference-normalized occupancies for each defined subunit in each of the 500 volumes from the 500 *k*-means cluster centers. The Jupyter notebook generated from the occupancy analysis scripts was used to perform hierarchical clustering with a row threshold of 0.75, which gave 24 classes. Most classes had high or full occupancy of $\alpha_2\varepsilon_2$ whereas only a few classes had medium or high occupancy of one or two β subunits. The filtering from occupancy Jupyter notebook was used to extract classes from clustering, which were cross-referenced against the star file that was used for the cryoDRGN input to generate star files for the desired classes that had medium or high occupancy of one or two β subunits (CODH/ACS pentamer or hexamer, respectively). Classes containing high occupancy of one β subunit were joined (CODH/ACS pentamer, 59,079 particles total) and separately, classes containing high occupancy of two β subunits were joined (CODH/ACS hexamer, 9,320 particles total) (bottom right of **Figure S8**). Each selection of particles was subjected to 3D refinement in RELION.

Now that the selections of CODH, CODH/ACS pentamer, and CODH/ACS hexamer were separated, they were subjected to final curation and refinements (**Figure S9**). All particles from sorting in RELION and cryoDRGN with downstream use of MAVEn were imported to cryoSPARC for removal of duplicates. After removal of duplicates, 17,073 remaining hexamer particles were subjected to homogeneous refinement with C2 symmetry (**Figure S9**). After removing duplicate pentamer particles (and removing any duplicate particles present in the hexamer particle stack), 66,930 particles remained and were subjected to homogeneous refinement. For the pentamer, 3D variability analysis (3DVA) was performed. Only frames from 3DVA display corresponding to fully intact pentamer were kept, leaving 33,375 particles then subjected to homogeneous refinement (**Figure S9**). Since the 136,062 CODH tetramer particles were selected from RELION 3D classification, there was a possibility that some of those particles were duplicates from the pentamer and/or hexamer particle stacks selected from the same pool of particles in cryoDRGN. Therefore, duplicates were also removed from the CODH tetramer particle stack, leaving 132,589 CODH tetramer particles, which were subjected to homogeneous refinement with C2 symmetry (**Figure S9**). Each particle stack (tetramer, pentamer, hexamer) was put through the pyem v0.5 software csparc2star to generate a RELION star file. For the tetramer, particles were re-extracted in RELION and a new initial model was generated without symmetry. Particles were subjected to a final round of 3D classification with two classes. The best class was selected, containing 125,759 particles. These particles were used to generate a new initial model with C2 symmetry. From this initial model forward, C2 symmetry was applied in all jobs for the tetramer. This model was subjected to 3D refinement,

Bayesian polishing, and per-particle CTF refinement before a final round of 3D refinement with a reference mask and postprocessing in RELION to yield a final map with global resolution of 2.8 Å (bottom of **Figure S9, Table S1**). Fourier shell correlation (FSC) plot, angular distribution plot, 3D FSC plot generated using the 3D FSC web server, and local resolution for the tetramer reconstruction are presented in **Figure S10**. For the pentamer, particles were re-extracted in RELION and subjected to 3D refinement without a reference mask prior to another round of 3D refinement with a reference mask. Next, particles were subjected to per-particle CTF refinement and Bayesian polishing before a final round of 3D refinement with a reference mask and postprocessing in RELION to yield a final map with global resolution of 3.2 Å (**Figure S9, Table S1**). Fourier shell correlation plot, angular distribution plot, 3D FSC plot generated using the 3D FSC web server, and local resolution for the pentamer reconstruction are presented in **Figure S11**. For the hexamer, particles were re-extracted in RELION. For all subsequent steps, C2 symmetry was applied. Hexamer particles were subjected to 3D refinement without a reference mask prior to another round of 3D refinement with a reference mask. Next, particles were subjected to per-particle CTF refinement and Bayesian polishing before a final round of 3D refinement with a reference mask and postprocessing in RELION to yield a final map with global resolution of 3.2 Å (**Figure S9, Table S1**). Fourier shell correlation (FSC) plot, angular distribution plot, 3D FSC plot generated using the 3D FSC web server, and local resolution for the hexamer reconstruction are presented in **Figure S12**.

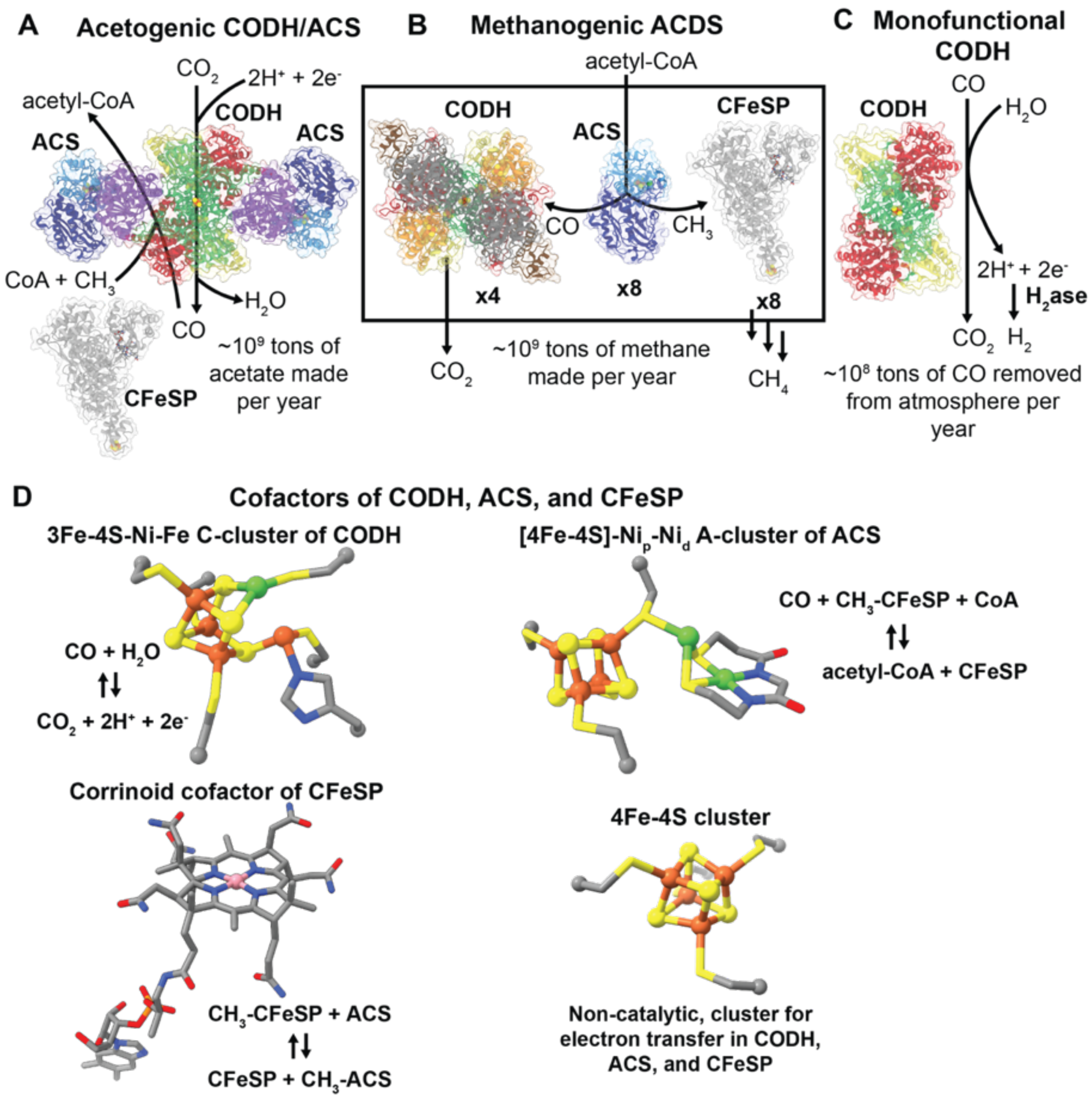

Supplementary Figure 1: CODHs contribute to the carbon cycle through CO oxidation, acetogenesis, and methanogenesis using complex metallocofactors. A. Acetogenic CODH/ACS performs acetyl-CoA synthesis and interacts transiently with CFeSP (PDB IDs: 1MJG, 4DJF). **B.** In methanogens, CODH (PDB ID: 3CF4), ACS, and CFeSP form a higher order stable complex. The structures of methanogenic ACS and CFeSP have not been solved previously, therefore structures of their acetogenic homologs are shown (PDB IDs: 1MJG, 4DJF), with A1 of ACS hidden. For methanogenesis, ACDS performs the disproportionation of acetyl-CoA into CO and a methyl moiety. **C.** Monofunctional CODH performs CO oxidation (PDB ID: 1JQK). CODH and ACS are colored by domains defined in Figure 2. **D.** Cofactors of CODH, ACS, and CFeSP. From left to right: [3Fe-4S-Ni-Fe] C-cluster of CODH, [4Fe-4S]-Ni_p-Ni_d A-cluster of ACS, corrinoid cofactor of CFeSP, and 4Fe-4S cluster used by CODH, ACS, and CFeSP. Atoms are colored: Ni green; Fe orange; S yellow; C grey; O red; N blue.

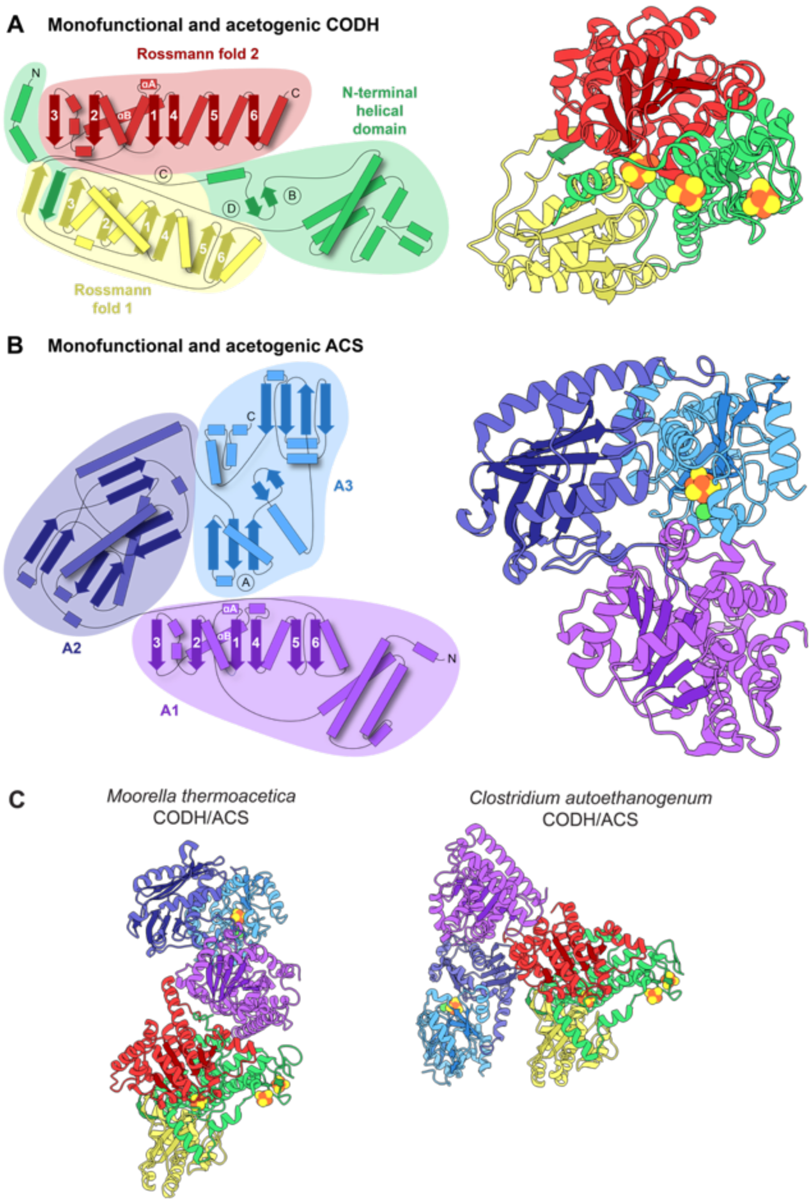

Supplementary Figure 2. Topology and ribbon diagrams of monofunctional and acetogenic CODH and ACS. A. Left: Topology of one protomer of the monofunctional and acetogenic CODH based on structures from *Rhodospirillum rubrum* (1JQK), *Carboxydothermus hydrogenoformans* (1SU8, 6ELQ, 7ZKJ), *Desulfovibrio vulgaris* (6B6V), *Moorella thermoacetica* (1MJG), and *Clostridium autoethanogenum* (6YTT). Right: Ribbon drawing of one protomer of the monofunctional CODH (from *R. rubrum*, 1JQK). Cluster positions are indicated as A, B, C, D for the A-cluster, B-cluster, C-cluster, and D-cluster, respectively. The six parallel strands associated with a classic Rossmann fold are labeled 1-6. Helices αA and αB mentioned in the text are labelled αA (before strand 1) and αB (between strands 3 and 4). **B.** Left: Topology of monofunctional and acetogenic ACS based on structures from *M. thermoacetica* (1MJG), *C. autoethanogenum* (6YTT), and *C. hydrogenoformans* (1RU3, 7ZKJ). Right: Ribbon drawing of an acetogenic ACS (from *M. thermoacetica*, 6X5K). Labels as described in A. **C.** CODH/ACS interface in *M. thermoacetica* (left) and *C. autoethanogenum* (right).

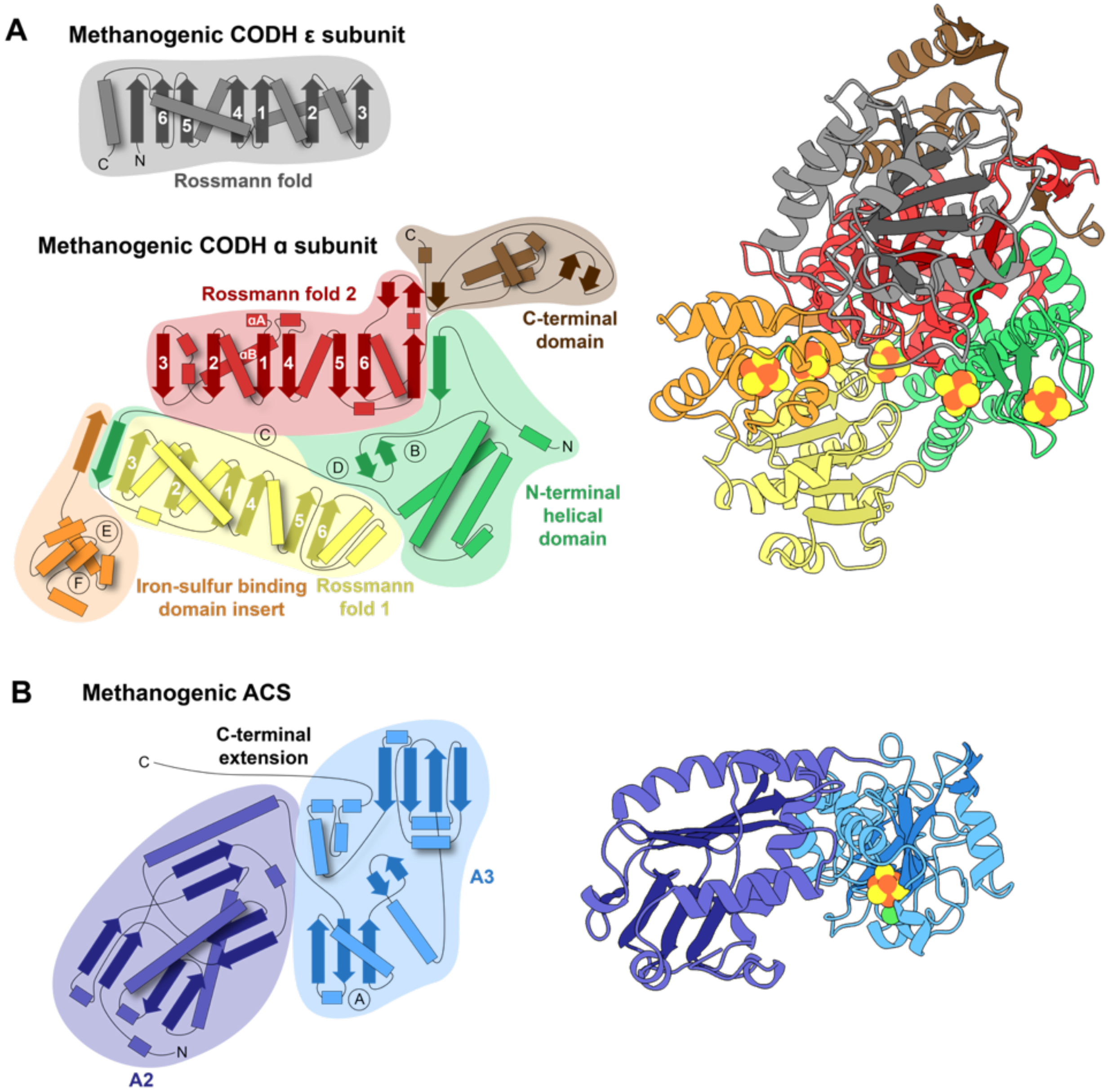

Supplementary Figure 3. Topology and ribbon diagrams for structures of methanogenic CODH and ACS. A. Left: Topology of methanogenic CODH α and ε subunit based on structures from *Methanosarcina barkeri* (3CF4) and *Methanosarcina thermophila* (this work). Right: Ribbon drawing of one of the two heterodimers of the heterotetrameric methanogenic CODH (from *M. thermophila*). **B.** Left: Topology of methanogenic ACS from *M. thermophila* (this work). Right: Ribbon drawing of a methanogenic ACS (from *M. thermophila*). In all panels, cluster positions are indicated as A, B, C, D, E, F for the A-cluster, B-cluster, C-cluster, D-cluster, E-cluster and F-cluster, respectively. The six parallel strands associated with a classic Rossmann fold are labeled 1-6. Helices αA and αB mentioned in the text are labelled αA (before strand 1) and αB (between strands 3 and 4).

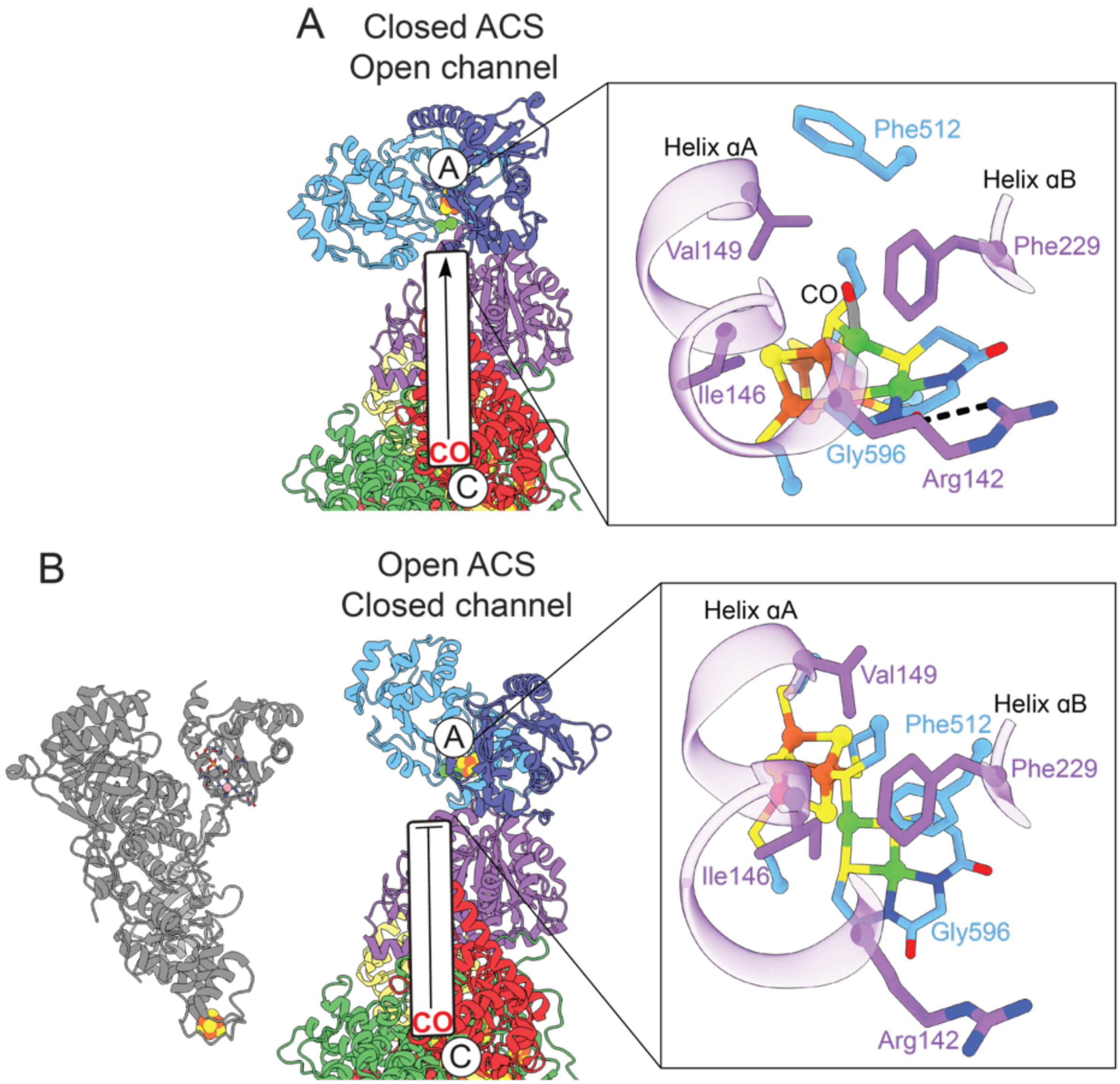

Supplementary Figure 4. Closed and open conformational states of ACS in *Moor*CODH/ACS. A. The closed ACS/open channel structure of *Moor*CODH/ACS was observed by crystallography (PDB ID: 6X5K). Inset: CO-binding alcove in the closed *Moor*ACS conformation, with helices αA and αB labeled. **B.** The open ACS/closed channel structure of *Moor*CODH/ACS was observed by crystallography (PDB ID: 1OAO). Inset: Movement of alcove residues and helix αA in the open *Moor*ACS conformation. This conformation is *en route* to the methylation-competent state, wherein a methyl group is donated by CFeSP (grey). All structures are aligned with respect to the A1 domain of ACS. ACS A1 is colored in purple, A2 is colored in dark blue, and A3 is colored in light blue. CODH N-terminal helical domain is colored in green, Rossmann fold 1 is colored in yellow, and Rossmann fold 2 is colored in red. Atoms are colored: Ni green; Fe orange; S yellow.

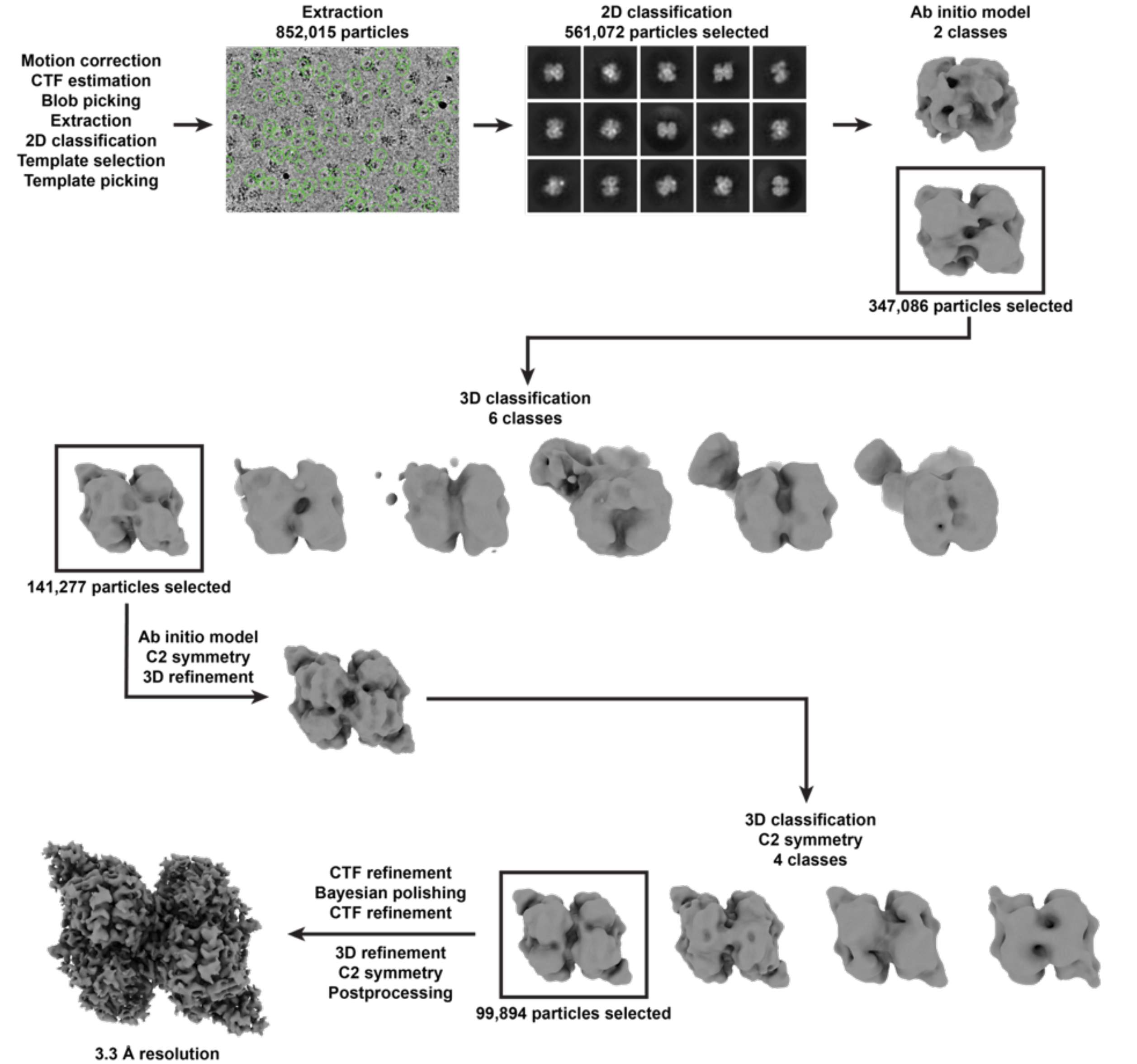

Supplementary Figure 5. Data processing workflow for Cp3-plunged sample. Preprocessing was completed in cryoSPARC, giving 852,015 extracted particles after template picking. Particles were curated through 2D classification and *ab initio* model generation in cryoSPARC, followed by 3D classification in RELION, yielding a final selection of 99,894 particles. After all refinements, a global resolution of 3.3 Å was achieved.

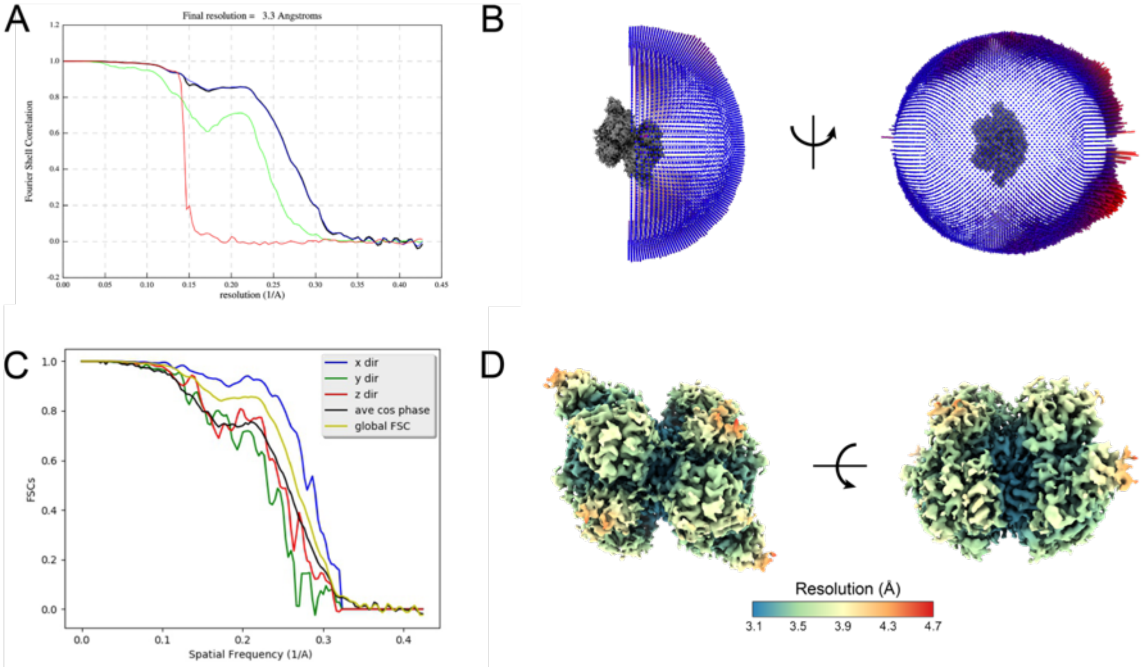

Supplementary Figure 6. Fourier shell correlation (FSC) curves, preferred orientation analysis, and local resolution for Cp3-plunged CODH tetramer. A. FSC curves: corrected (black), unmasked (green), masked (blue), and phase-randomized masked (red). Resolution indicated at the top of each plot is the resolution at 0.143 FSC on the corrected map. Plot was generated using RELION. **B.** Angular distribution plot generated from RELION 3D autorefine. **C.** 3D FSC plots indicating the Fourier shell correlation as a function of spatial frequency in the x-direction (blue), y-direction (green), and z-direction (red), as well as the overall global FSC curve (goldenrod) and the average cosine phase (black). Plots were generated using the 3DFSC web server (3dfsc.salk.edu). **D.** Postprocessed map colored by resolution.

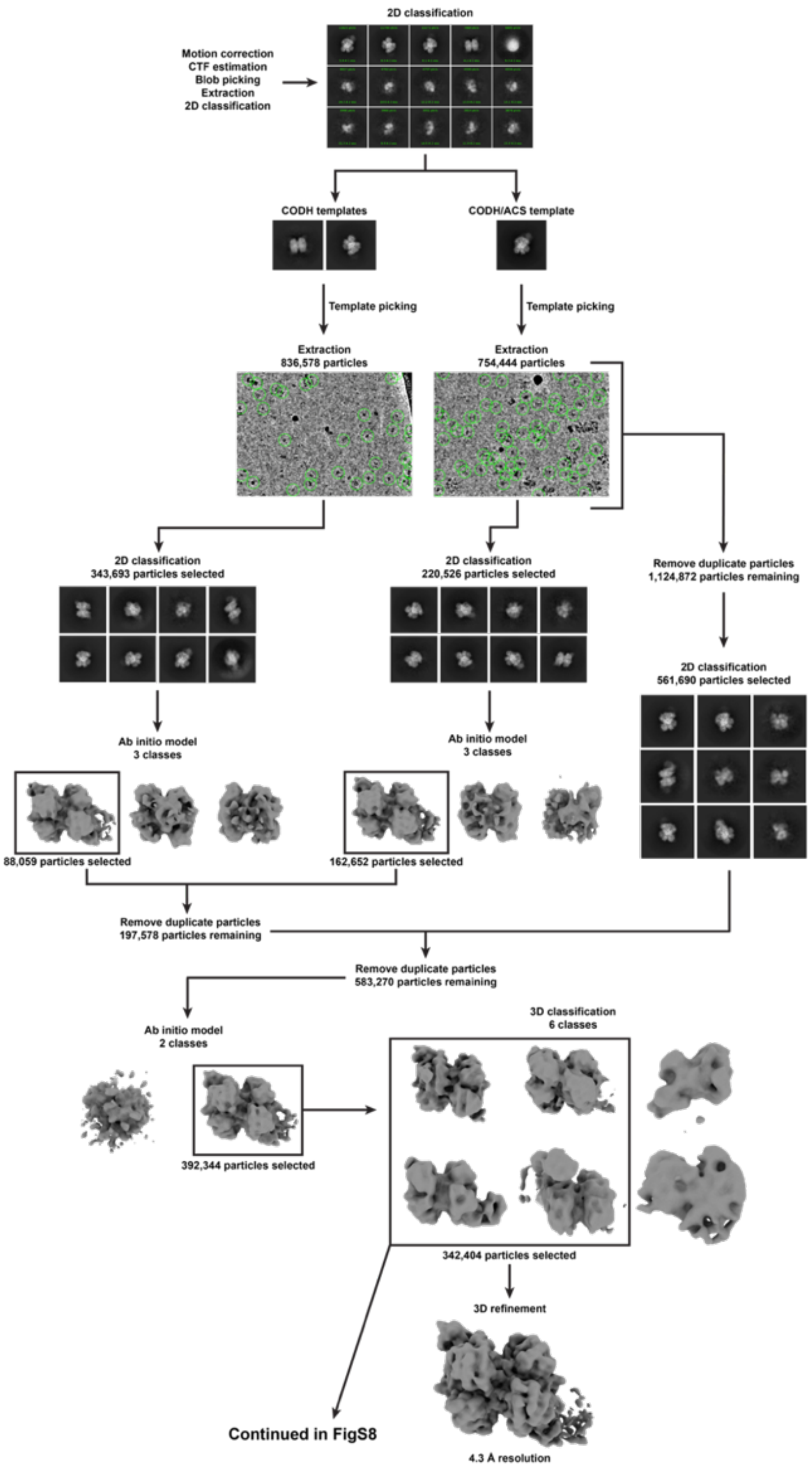

Supplementary Figure 7. Preprocessing and initial particle selection for chameleon-plunged sample. These steps were completed in cryoSPARC. After ab initio model generation, one round of 3D classification was completed in RELION to filter out junk and denatured particles. The selection of 342,404 particles refined to 4.3 Å resolution and contained a mixture of CODH, CODH/ACS pentamer, and CODH/ACS hexamer.

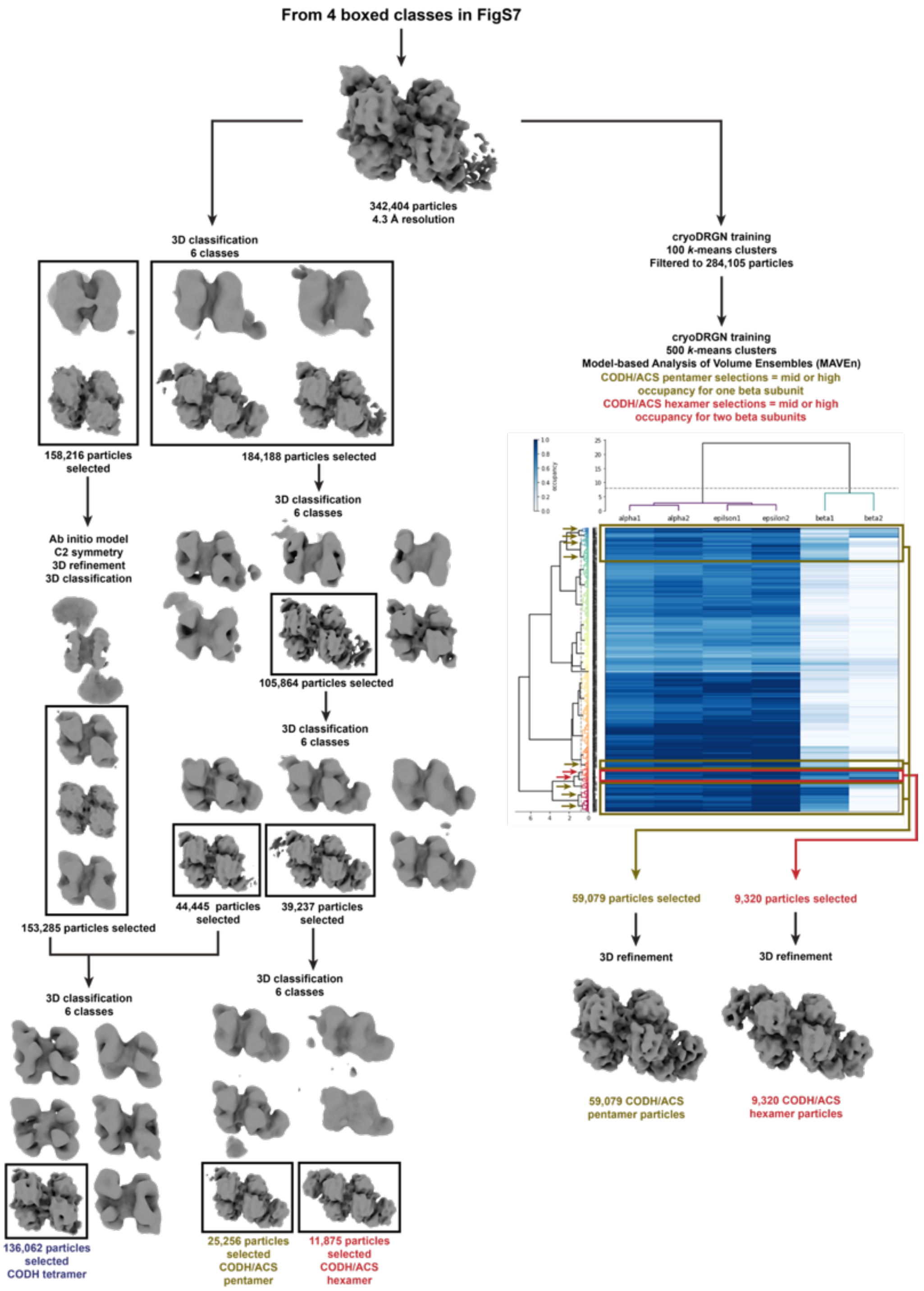

Supplementary Figure 8. Separation of oligomers in chameleon-plunged sample. In parallel, separation of CODH, CODH/ACS pentamer, and CODH/ACS hexamer was completed using RELION 3D classification and cryoDRGN with the downstream MAVEn tool. From RELION, 136,062 tetramer particles were selected, 25,256 pentamer particles were selected, and 11,875 hexamer particles were selected. From cryoDRGN and MAVEn, 59,079 pentamer particles and 9,320 hexamer particles were selected. Color coding for lines and text: tetramer in dark blue, pentamer in mustard, hexamer in red.

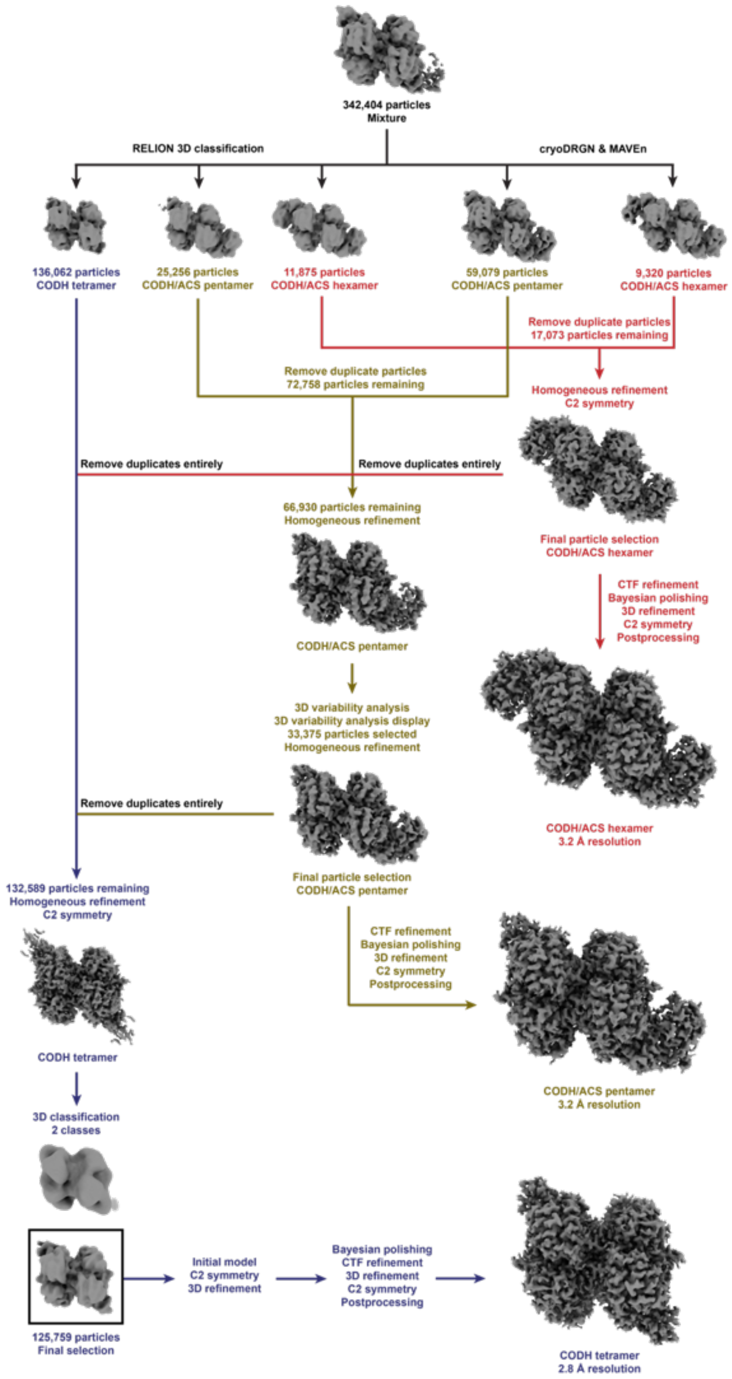

Supplementary Figure 9. Final particle curation and refinements for chameleon-plunged sample. This final particle curation was completed using removal of duplicate particles in cryoSPARC along with filtering of tetramer particles through 3D classification in RELION and of pentamer particles through 3DVA in cryoSPARC. In total, 125,759 tetramer particles were selected, 33,375 pentamer particles were selected, and 17,073 hexamer particles were selected. CTF refinements, Bayesian polishing, 3D refinements, and postprocessing were completed in RELION, yielding global resolutions of 2.8, 3.2, and 3.2 Å for the tetramer, pentamer, and hexamer, respectively. Color coding for lines and text: tetramer in dark blue, pentamer in mustard, hexamer in red.

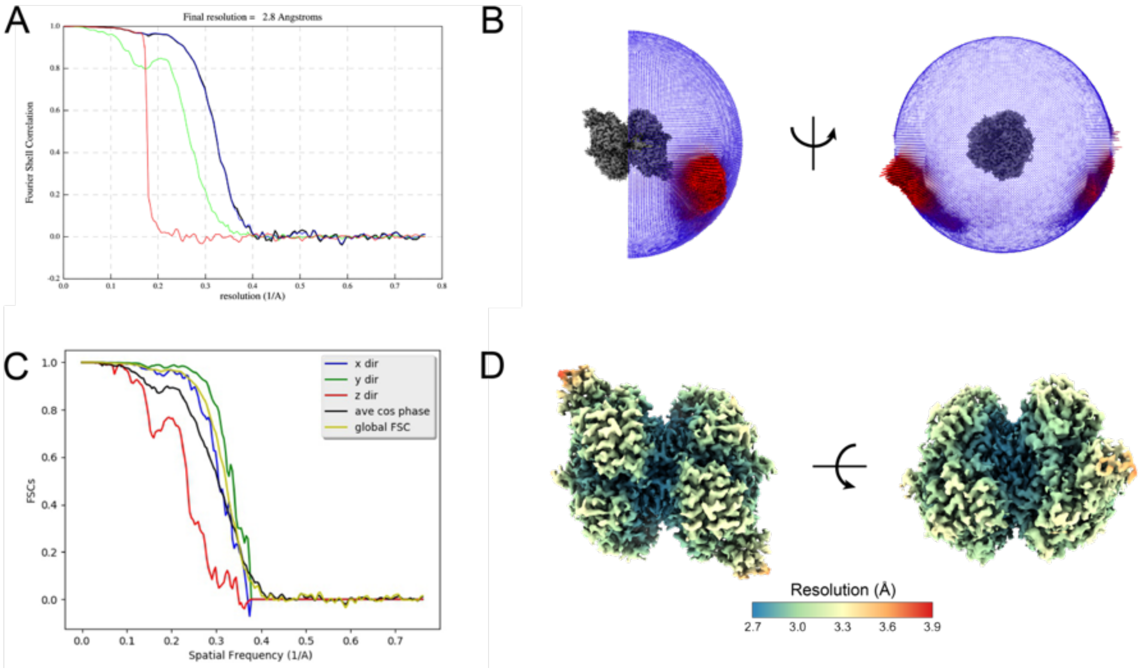

Supplementary Figure 10. Fourier shell correlation (FSC) curves, preferred orientation analysis, and local resolution for chameleon-plunged CODH tetramer. A. FSC curves: corrected (black), unmasked (green), masked (blue), and phase-randomized masked (red). Resolution indicated at the top of each plot is the resolution at 0.143 FSC on the corrected map. Plot was generated using RELION. **B.** Angular distribution plot generated from RELION 3D autorefine. **C.** 3D FSC plots indicating the Fourier shell correlation as a function of spatial frequency in the x-direction (blue), y-direction (green), and z-direction (red), as well as the overall global FSC curve (goldenrod) and the average cosine phase (black). Plots were generated using the 3DFSC web server (3dfsc.salk.edu). **D.** Postprocessed map colored by resolution.

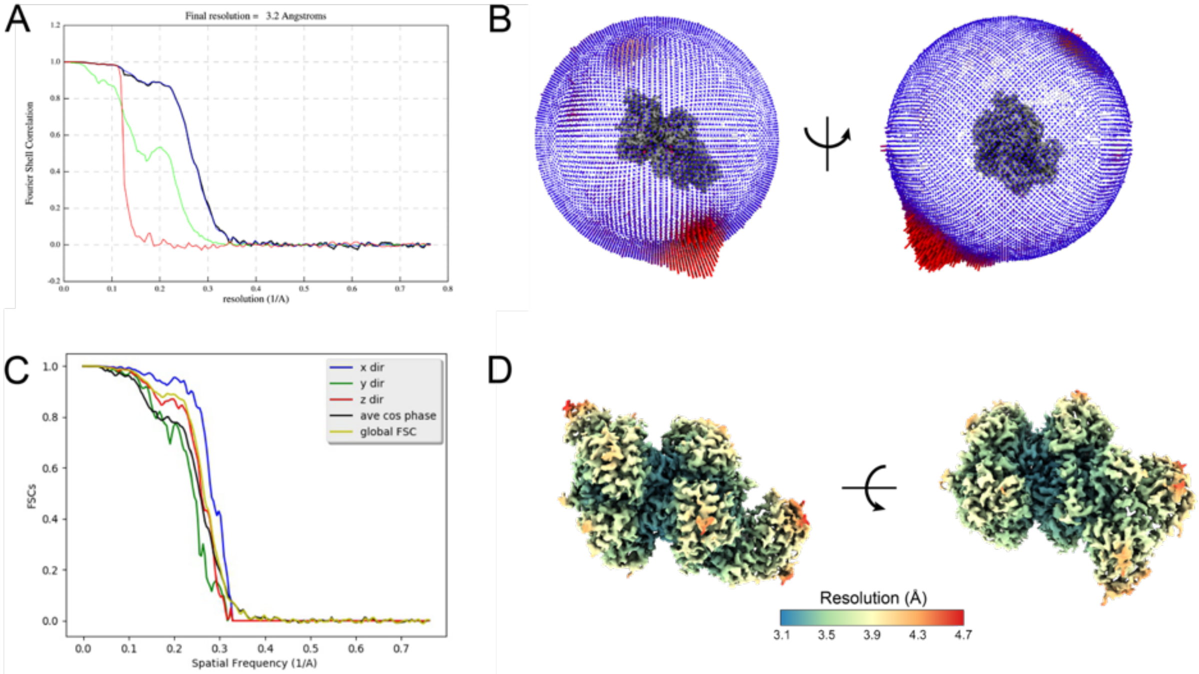

Supplementary Figure 11. Fourier shell correlation (FSC) curves, preferred orientation analysis, and local resolution for CODH/ACS pentamer. A. FSC curves: corrected (black), unmasked (green), masked (blue), and phase-randomized masked (red). Resolution indicated at the top of each plot is the resolution at 0.143 FSC on the corrected map. Plot was generated using RELION. **B.** Angular distribution plot generated from RELION 3D autorefine. **C.** 3D FSC plots indicating the Fourier shell correlation as a function of spatial frequency in the x-direction (blue), y-direction (green), and z-direction (red), as well as the overall global FSC curve (goldenrod) and the average cosine phase (black). Plots were generated using the 3DFSC web server (3dfsc.salk.edu). **D.** Postprocessed map colored by resolution.

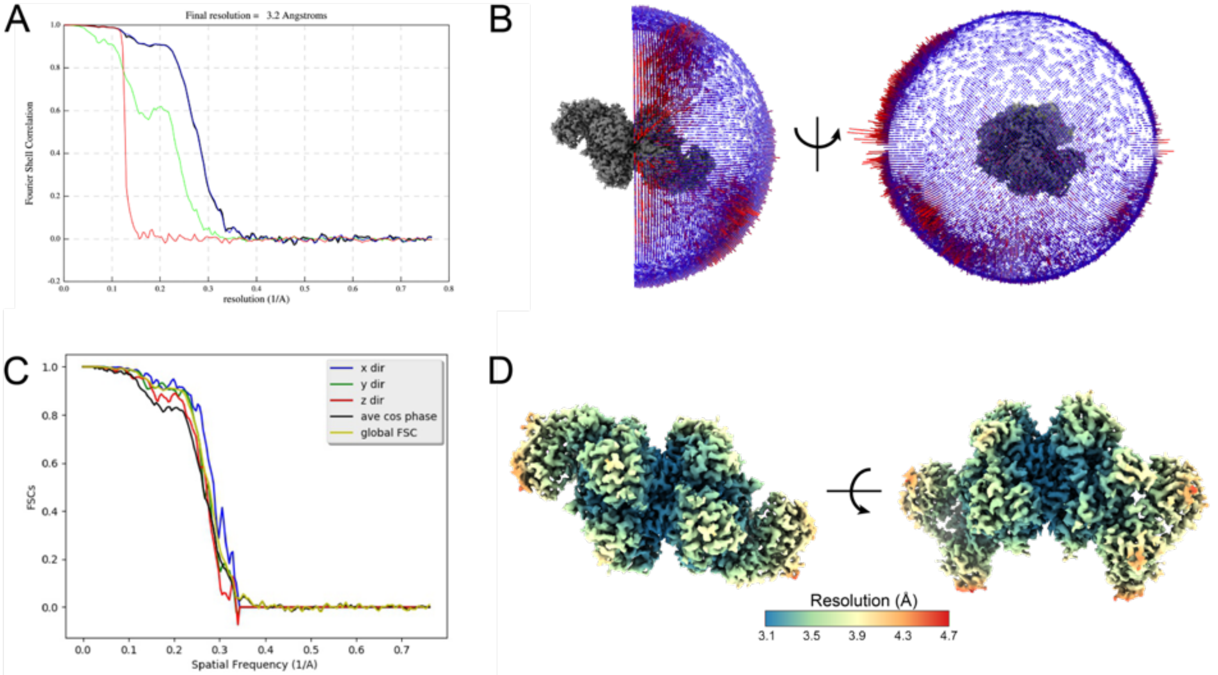

Supplementary Figure 12. Fourier shell correlation (FSC) curves, preferred orientation analysis, and local resolution for CODH/ACS hexamer. A. FSC curves: corrected (black), unmasked (green), masked (blue), and phase-randomized masked (red). Resolution indicated at the top of each plot is the resolution at 0.143 FSC on the corrected map. Plot was generated using RELION. **B.** Angular distribution plot generated from RELION 3D autorefine. **C.** 3D FSC plots indicating the Fourier shell correlation as a function of spatial frequency in the x-direction (blue), y-direction (green), and z-direction (red), as well as the overall global FSC curve (goldenrod) and the average cosine phase (black). Plots were generated using the 3DFSC web server (3dfsc.salk.edu). **D.** Postprocessed map colored by resolution.

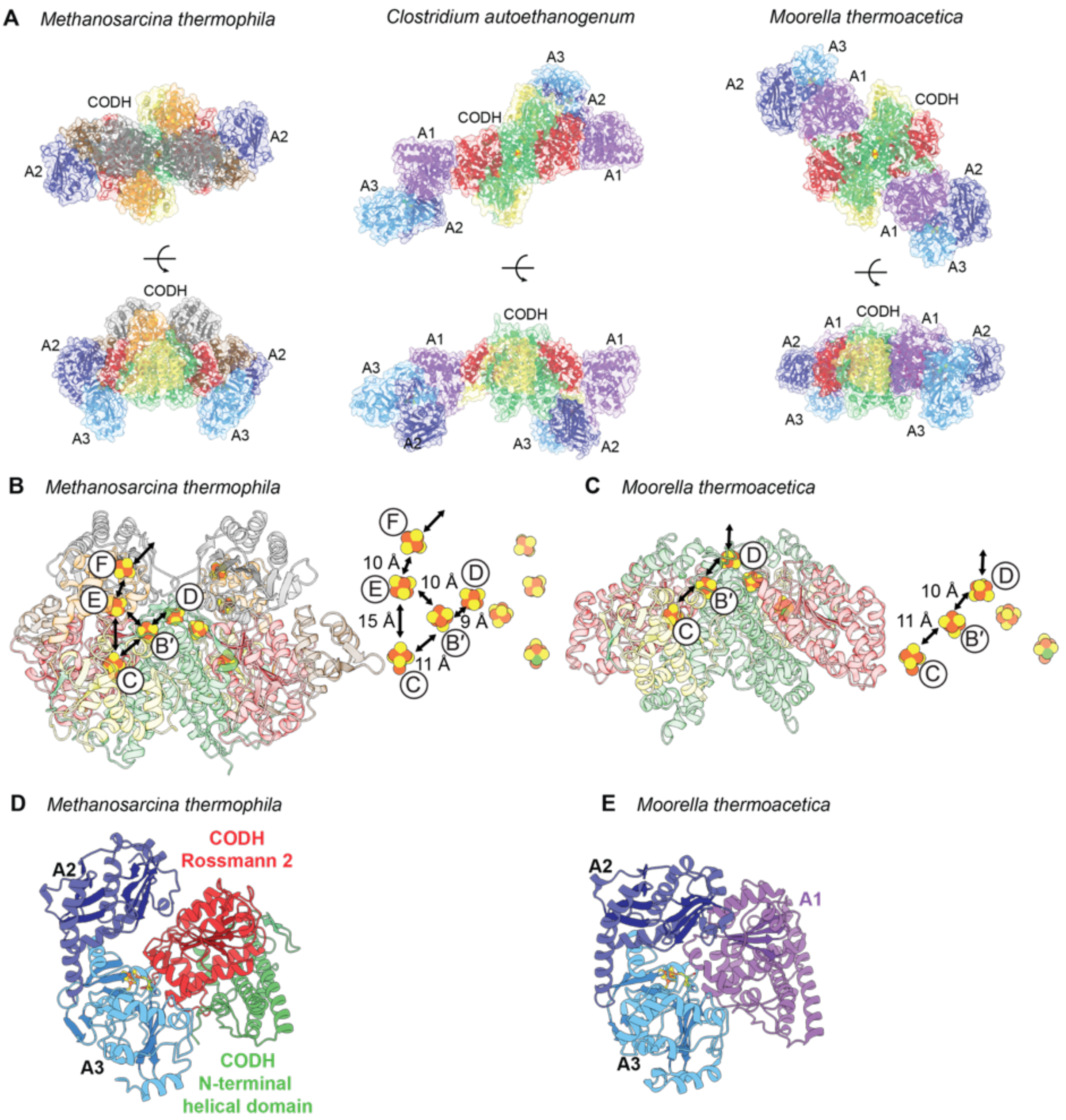

Supplementary Figure 13. Comparison of CODHs and ACSs in methanogens and acetogens. Colored as in Figure 2. **A.** Comparison of CODH/ACS complexes. Left: Methanogenic CODH/ACS from *M. thermophila* (this work). Middle: Acetogenic CODH/ACS from *C. autoethanogenum* (PDBID: 6YTT). Right: Acetogenic CODH/ACS from *M. thermoacetica* (PDBID: 6X5K). **B.** Possible pathways for electrons from the buried C-cluster to the solvent exposed F-cluster in *Met*CODH. **C.** Pathway for electrons from the buried C-cluster to the solvent exposed D-cluster in *Moor*CODH. Distances shown are nearest atom to nearest atom. **D.** *Met*CODH (red & green) in complex with *Met*ACS (A2 in cyan & A3 in blue). **E.** *Moor*ACS (PDBID: 6X5K). A1 in purple, A2 in cyan, A3 in blue. Atoms are colored: Ni green; Fe orange; S yellow; CO grey/red; O red; N blue.

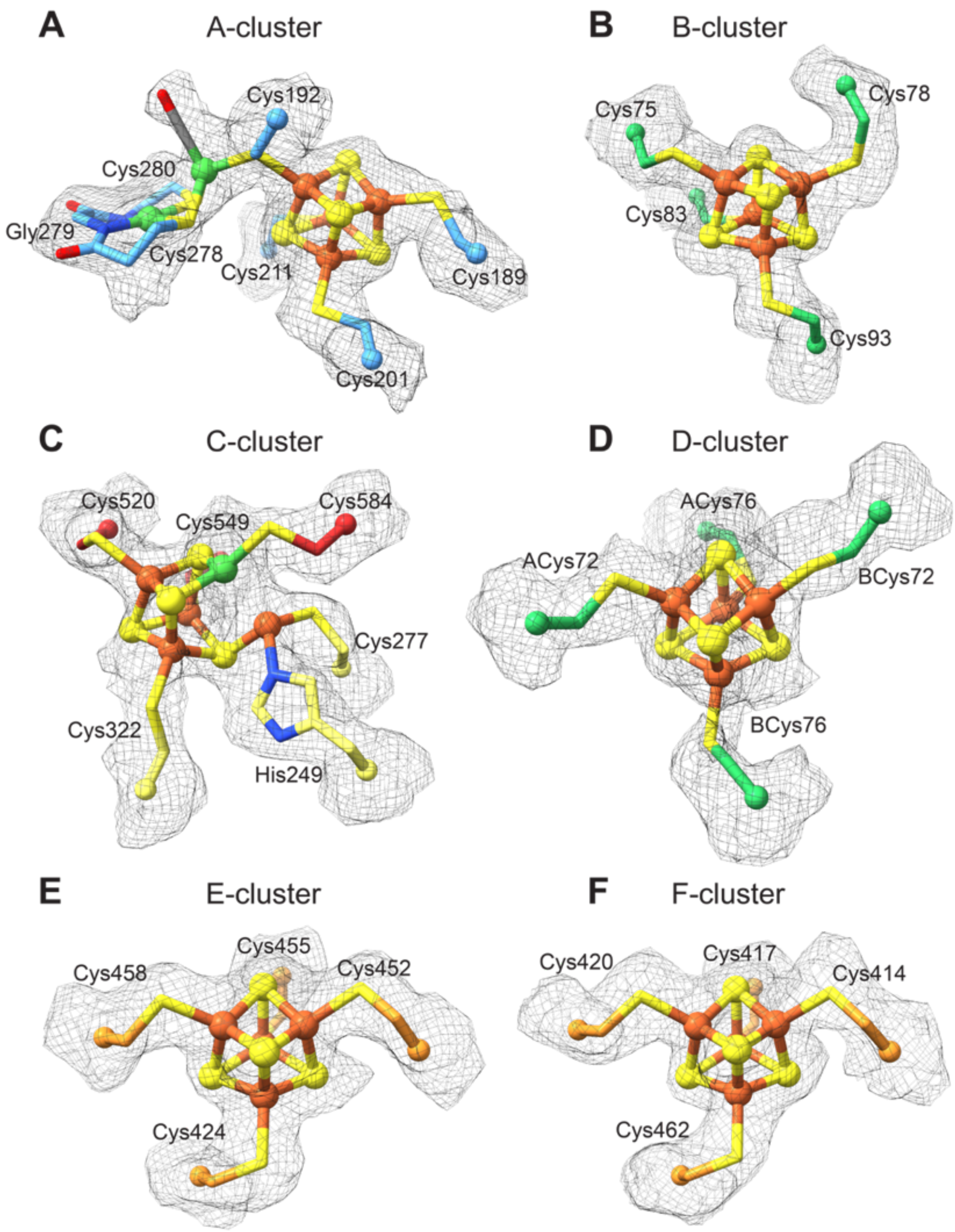

Supplementary Figure 14. Cryo-EM density for metallocofactors of CODH and ACS. A. A-cluster density from the CODH/ACS hexamer. **B.** B-cluster density from chameleon-plunged CODH. **C.** C-cluster density from chameleon-plunged CODH. **D.** D-cluster density from chameleon-plunged CODH. **E.** E-cluster density from chameleon-plunged CODH. **F.** F-cluster density from chameleon-plunged CODH. Ligating residues are shown. Atoms are colored: Ni green; Fe orange; S yellow; CO grey/red; O red; N blue. Carbons are labeled by color of domain in Fig. 2.

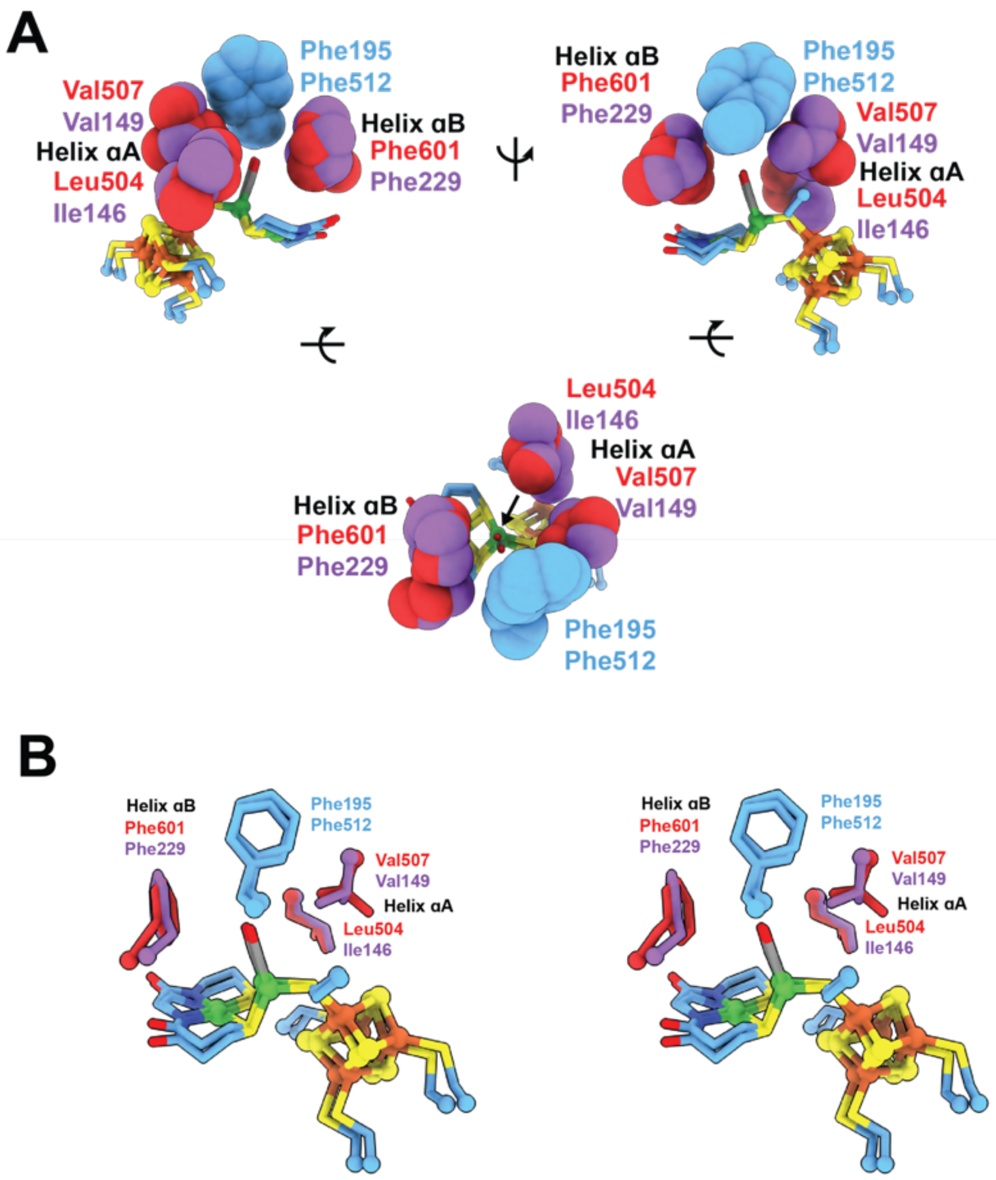

Supplementary Figure 15. CO alcove in methanogenic CODH/ACS. A. CO alcove at the A-cluster of *Met*CODH/ACS and *Moor*ACS, with alcove residues depicted in a space-filling representation. **B.** Stereo view of the hydrophobic CO binding alcove at the A-cluster. The *Met*CODH/ACS and *Moor*ACS are overlaid. The A-cluster is colored: Ni green; Fe orange; S yellow; CO grey/red. Carbons colored by domains as in Figure 2.

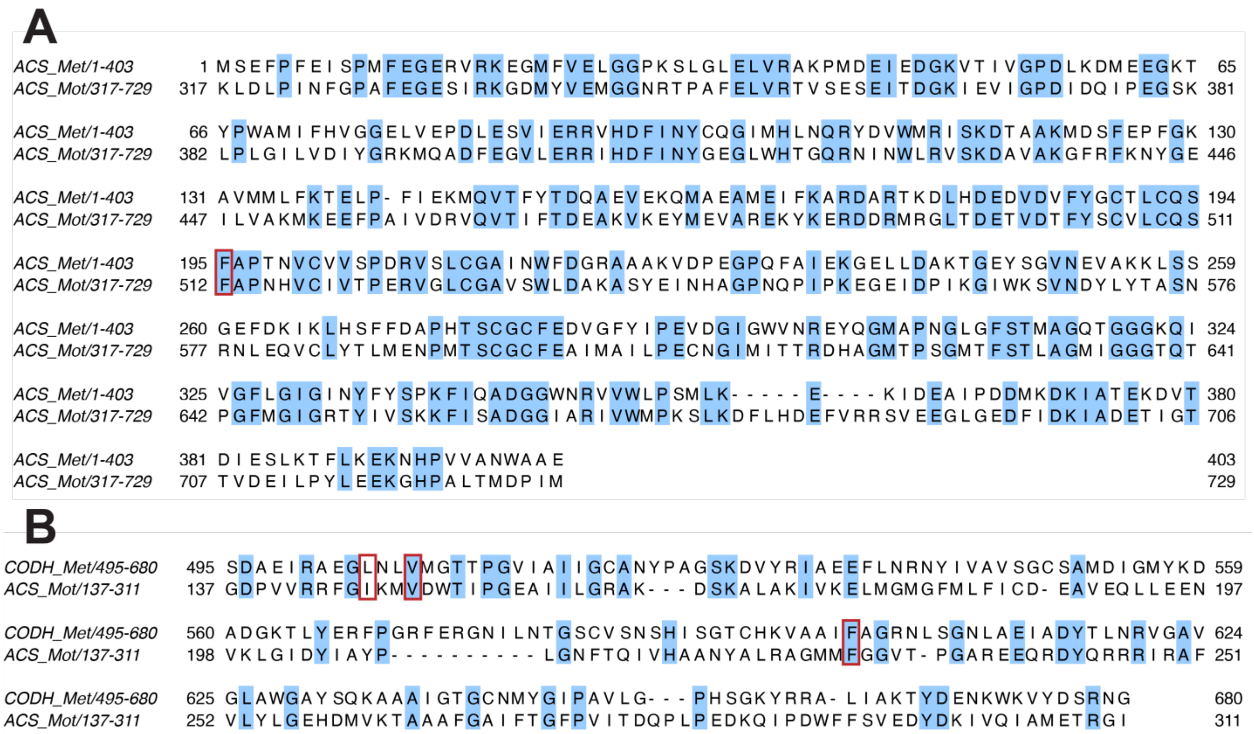

Supplementary Figure 16. Sequence alignments of *Moor*ACS with *Met*ACS and *Met*CODH. A. Pairwise alignment of the sequences of A2 and A3 of *Met*ACS and *Moor*ACS. Alcove residue Phe195(*Met*)/Phe512(*Moor*) is indicated with a maroon box. **B.** Pairwise alignment of the sequences of Rossmann domain 2 of *Met*CODH and the A1 Rossmann domain of *Moor*ACS. Alcove residues Leu504(*Met*)/Ile146(*Moor*), Val507(*Met*)/Val149(*Moor*), and Phe601(*Met*)/Phe229(*Moor*) are indicated with maroon boxes. Conserved residues are colored in blue.

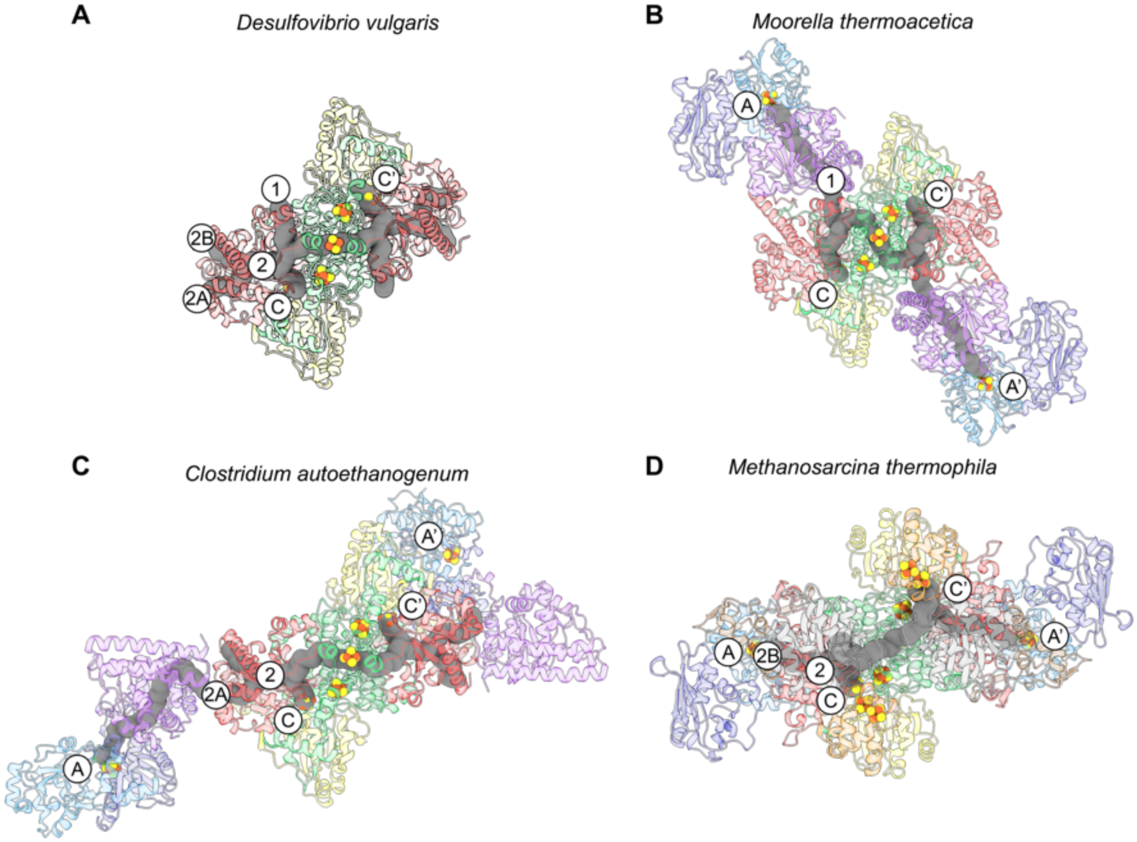

Supplementary Figure 17. Calculated cavities in CODH and CODH/ACS enzymes. A. The monofunctional CODH from *D. vulgaris* has three gas channels (1, 2A and 2B) elucidated by xenon studies (PDBID: 7TSJ). Here, the structure of *D. vulgaris* CODH is shown with its calculated cavities. **B.** Structure of *M. thermoacetica* CODH/ACS (PDBID: 6X5K) with calculated cavities. **C.** Structure of *C. autoethanogenum* CODH/ACS (PDBID: 6YTT) with calculated cavities. **D.** Structure of *M. thermophila* CODH/ACS (this work) with calculated cavities. Cavities were calculated using MOLEonline, and calculated cavities are shown in grey. Proteins colored as in Figure 2. See Figure 4 for comparison of calculated cavities with xenon-identified gas channels.

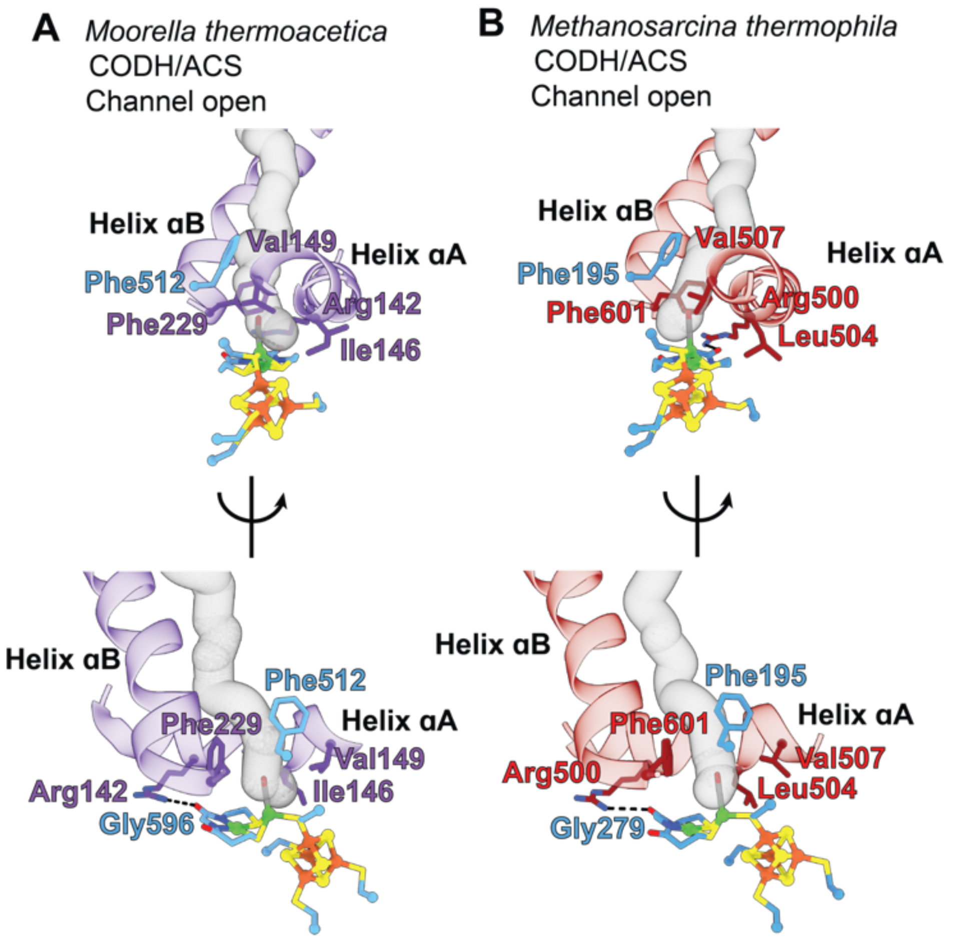

Supplementary Figure 18. Conserved Rossmann helices form the gas channel in acetogens and methanogens. A. In the open-channel/closed-ACS conformation of *Moor*ACS (PDBID: 6X5K), the gas channel passes between helices αA and αB, with alcove residues surrounding the CO site and Arg142 is interacting with A-cluster coordinating residue (Gly596). **B.** In *Met*CODH/ACS, the gas channel also passes between helices αA and αB, with alcove residues surrounding the CO site and Arg500 is interacting with A-cluster coordinating residue (Gly279).

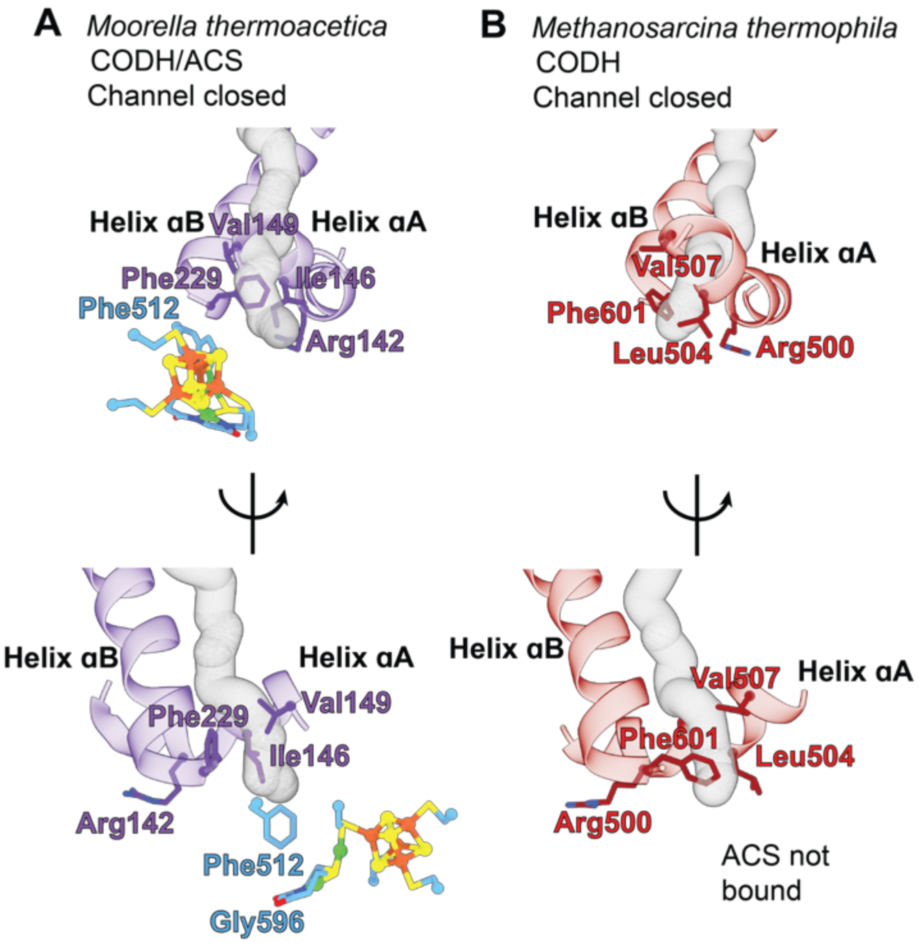

Supplementary Figure 19. Rearrangement of conserved Rossmann helices and alcove residues caps the gas channel in acetogens and methanogens. A. In the closed-channel/open-ACS conformation of *Moor*ACS (PDBID: 1OAO), helix αA blocks the gas channel, Phe512 blocks the CO binding site at the A-cluster, and Arg142 has swung away from the A-cluster breaking its hydrogen bond. **B.** In *Met*CODH (ACS not bound), helix αA shifts to block the gas channel, with Phe601 also swinging in to block the channel.

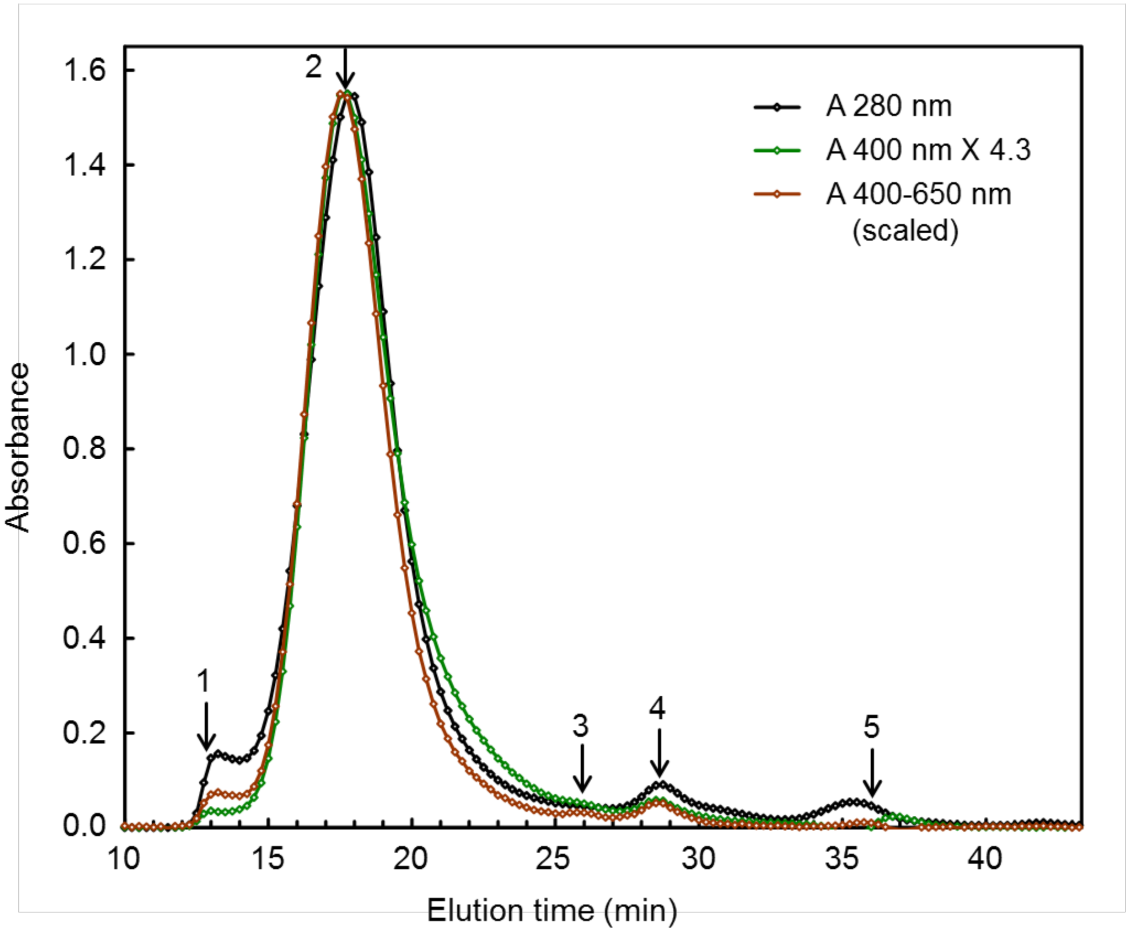

Supplementary Figure 20. Analysis of ACDS preparation by SEC on Superose 6 HR 10/30. A sample of the ACDS preparation was thawed, adjusted to contain 0.1 M Na_2SO_4, and 0.26 ml was applied to a 1.0 X 30 cm Superose 6 HR column. Flow rate was 0.60 ml/min using 50 mM MOPS buffer, 0.1 M Na_2SO_4, pH 7.2. UV-visible absorbance spectra were recorded on the effluent at 15 s intervals using an HP 8452A spectrophotometer with an 80 µl black-walled flow cell. All steps were performed under anaerobic conditions inside a Coy type anaerobic chamber. Arrows indicate the positions of; 1. column void volume, V_0, MW >5 MDa, 2. ACDS peak, 3. MW 220 kDa $\alpha_2\varepsilon_2$ CODH, 4. unknown contaminant, 5. column total volume, V_t, non-interacting salts. The results indicate good homogeneity on both sides of the main peak in the region of ~16-20 min. Contamination by non-ACDS protein is indicated on the low molecular mass side in the region of 20-25 min and at around 28.5 min (arrow 4). None of the 5 ACDS subunits were found at the void volume. Re-chromatography of the main peak (not shown) produced a peak at the same position with very close to the same width at half height as found originally, but with contaminants mostly eliminated, indicating that under these conditions the ACDS subunits remain associated, with very little amount of dissociation into smaller sub-complexes.

Supplementary Table 1. Cryo-EM data collection, refinement, and validation statistics

Datasets	Cp3-plunged	Chameleon-plunged		
**Structure determined**	CODH tetramer	CODH tetramer	CODH/ACS pentamer	CODH/ACS hexamer
**PDB ID**	9C0Q	9C0S	9C0R	9C0T
**EMDB ID**	EMD-45089	EMD-45090	EMD-45091	EMD-45092
**Microscope**	Titan Krios	Titan Krios		
**Camera**	Gatan K3	Gatan K3		
**Magnification**	105,000x	130,000x		
**Voltage (kV)**	300	300		
**Recording mode**	counting	counting		
**Frames/Movies**	21	40		
**Total Electron dose (e-/Å2)**	54.53	47.1		
**Defocus range (μm)**	-0.8 to -2.1	0 to -1.75		
**Pixel size (Å)**	0.832	0.654		
**Micrographs collected**	14,078	13,892		
**Automation software**	EPU	EPU		
**Total extracted particles**	852,015	1,124,872		
**Refined particles**	99,894	125,759	33,375	17,073
**Symmetry imposed**	C2	C2	C1	C2
**Map sharpening B-factor**	-90.5204	-74.1561	-76.1221	-67.9504
**Nominal Map Resolution (Å)**	3.3	2.8	3.2	3.2
**FSC threshold**	0.143	0.143	0.143	0.143
**masked/unmasked**	3.3 / 3.7	2.8 / 3.2	3.2 / 3.9	3.2 / 3.7
**Local resolution range (Å)**	3.1 – 7.3	2.7 – 4.4	3.1 – 5.2	3.0 – 5.4
**Refinement**				
**Number of atoms**				
**Protein**	14,188	14,342	17,384	20,514
**A-cluster**	0	0	10	20
**B-cluster**	16	16	16	16
**C-cluster**	18	18	18	18
**D-cluster**	8	8	15*	8
**E-cluster**	16	16	16	16

**F-cluster**	16	16	16	16
**Carbon monoxide**	0	0	2	4
**Water**	0	316	0	0
**Residues**				
**Chain A (subunit α)**	40-798 (of 803)	40-801 (of 803)	40-801 (of 803)	39-801 (of 803)
**Chain B (subunit α)**	40-798 (of 803)	40-801 (of 803)	40-800 (of 803)	39-801 (of 803)
**Chain C (subunit ε)**	4-170 (of 170)	3-170 (of 170)	2-170 (of 170)	1-170 (of 170)
**Chain D (subunit ε)**	4-170 (of 170)	3-170 (of 170)	2-170 (of 170)	1-170 (of 170)
**Chain E (subunit β)**			5-401 (of 472)	4-396 (of 472)
**Chain F (subunit β)**				4-396 (of 472)
**MapCC (mask/box)**	0.86 / 0.76	0.85 / 0.77	0.83 / 0.74	0.86 / 0.76
**Mean CC for ligands**	0.76	0.64	0.86	0.90
**R.m.s deviations**				
**Bond lengths (Å)**	0.004	0.005	0.003	0.004
**Bond angles (°)**	0.679	0.651	0.577	0.612
**MolProbity score**	2.01	1.74	1.78	1.68
**Clashscore (all atom)**	8.64	6.56	7.61	7.78
**Rotamer outliers (%)**	2.29	2.00	1.66	1.18
**Ramachandran plot**				
**Favored (%)**	96.0	97.2	96.8	96.7
**Allowed (%)**	4.0	2.8	3.2	3.3
**Outliers (%)**	0.0	0.0	0.0	0.00
**C-beta outliers (%)**	0.00	0.00	0.00	0.00
**CaBLAM outliers (%)**	2.67	1.79	2.06	1.86
**EMRinger Score**	2.53	3.71	2.65	3.33

*D-cluster in the CODH/ACS pentamer is modeled as a mixture of 4Fe–4S and 3Fe–4S clusters.

Supplementary Table 2. Closed state of ACS correlates with geometry of proximal metal.*

Species	ACS state	Channel state	PDB ID	Chain	Proximal metal	Ligand modeled	Proximal metal geometry
Carboxydothermus hydrogenoformans	Open	Closed	1RU3	A	Ni	Water	Square planar
Moorella thermoacetica	Open	Closed	1OAO	D	Ni	Acetate	Square planar
Moorella thermoacetica	Closed	Open	1OAO	C	Zn	Sulfur oxide	Tetrahedral
Moorella thermoacetica	Closed	Open	1MJG	M, N, O, P	Cu	Acetyl	Tetrahedral
Moorella thermoacetica	Closed	Open	3I01	M, N, O, P	Cu	Acetyl	Tetrahedral
Moorella thermoacetica	Closed	Open	3I04	M, N, O, P	Cu	Acetyl	Tetrahedral
Moorella thermoacetica	Closed	Open	6X5K	M, N, P	Ni	Carbon monoxide	Tetrahedral
Carboxydothermus hydrogenoformans	Closed	Open	7ZKJ	B	Ni	Acetate	Tetrahedral
Carboxydothermus hydrogenoformans	Closed	Open	7ZKK	B	Ni	Acetate	Tetrahedral
Carboxydothermus hydrogenoformans	Closed	Open	7ZKV	B	Ni	Acetate	Tetrahedral

*Only structures of A-clusters with standard architectures are included. Standard architecture is defined as an A-cluster with a cysteine bridging the 4Fe–4S cluster to the proximal metal, which is bridged to the distal metal by two cysteines.

Supplementary Table 3. Hydrogen bonds between CODH (chain A) and ACS (chain E) in the CODH/ACS complex.

	CODH Chain A	Dist. [Å]*	ACS Chain E
**1**	Ala141 [O]	3.2	Ser312 [OG]
**2**	Ala602 [O]	3.6	Lys322 [NZ]
**3**	Glu729 [OE2]	2.4	Ser9 [OG]
**4**	Ser755 [OG]	3.5	Ile8 [O]
**5**	Arg575 [NH2]	3.7	Glu13 [OE2]
**6**	Asn716 [ND2]	3.7	Leu191 [O]
**7**	Ser790 [OG]	2.6	Gln193 [OE1]
**8**	Arg620 [NE]	2.8	Ser194 [O]
**9**	Asn716 [ND2]	3.5	Ser194 [OG]
**10**	Asn716 [N]	3.6	Ser194 [OG]
**11**	Tyr616 [OH]	2.4	Phe195 [O]
**12**	Arg500 [NH1]	3.7	Gly279 [O]
**13**	Ala497 [N]	3.9	Ser312 [OG]

* Interactions calculated using PDBe PISA v1.52

Supplementary Table 4. Salt bridges between CODH (chain A) and ACS (chain E) in the CODH/ACS complex.

	**CODH Chain A**	**Dist. [Å]***	**ACS Chain E**
**1**	Arg575 [NH2]	3.7	Glu13 [OE2]
**2**	His748 [NE2]	3.7	Asp226 [OD2]

* Interactions calculated using PDBe PISA v1.52

Supplementary Table 5. Solvent-accessible area and solvation energy* for the CODH/ACS complex.

	**ACS**		**CODH**	
**Chain**	E		A	
**Number of atoms**				
interface	182	5.9%	181	3.1%
surface	1670	53.9%	2927	50.3%
total	3099	100.0%	5822	100.0%
**Number of residues**				
interface	55	14.0%	54	7.1%
surface	364	92.6%	672	88.3%
total	393	100.0%	761	100.0%
**Solvent-accessible area, Å**				
interface	1698.7	9.5%	1662.3	5.9%
total	17926.8	100.0%	28284.6	100.0%
**Solvation energy, kcal/mol**				
isolated structure	-400.5	100.0%	-741.7	100.0%
gain on complex formation	-12.4	3.1%	-4.3	0.6%
average gain	-4.1	1.0%	-3.9	0.5%

* Calculated using PDBe PISA v1.52

SI References

1. Punjani, A.; Rubinstein, J. L.; Fleet, D. J.; Brubaker, M. A., cryoSPARC: algorithms for rapid unsupervised cryo-EM structure determination. *Nat. Methods* **2017,** *14* (3), 290-296.
2. Asarnow, D., Palovcak, E., Cheng, Y. *UCSF pyem v0.5*, Zenodo: 2019.
3. Kimanius, D.; Dong, L.; Sharov, G.; Nakane, T.; Scheres, S. H. W., New tools for automated cryo-EM single-particle analysis in RELION-4.0. *Biochem J.* **2021,** *478* (24), 4169-4185.
4. Zhong, E. D.; Bepler, T.; Berger, B.; Davis, J. H., CryoDRGN: reconstruction of heterogeneous cryo-EM structures using neural networks. *Nat. Methods* **2021,** *18* (2), 176-185.
5. Kinman, L. F.; Powell, B. M.; Zhong, E. D.; Berger, B.; Davis, J. H., Uncovering structural ensembles from single-particle cryo-EM data using cryoDRGN. *Nat. Protoc.* **2023,** *18* (2), 319-339.
6. Tan, Y. Z.; Baldwin, P. R.; Davis, J. H.; Williamson, J. R.; Potter, C. S.; Carragher, B.; Lyumkis, D., Addressing preferred specimen orientation in single-particle cryo-EM through tilting. *Nat. Methods* **2017,** *14* (8), 793-796.
7. Pettersen, E. F.; Goddard, T. D.; Huang, C. C.; Meng, E. C.; Couch, G. S.; Croll, T. I.; Morris, J. H.; Ferrin, T. E., UCSF ChimeraX: Structure visualization for researchers, educators, and developers. *Protein Sci.* **2021,** *30* (1), 70-82.
